# Comprehensive Evaluation of Models for Ammonia Binding to the Oxygen Evolving Complex of Photosystem II

**DOI:** 10.1021/acs.jpcb.3c06304

**Published:** 2024-02-01

**Authors:** Maria Drosou, Dimitrios A. Pantazis

**Affiliations:** † Max-Planck-Institut für Kohlenforschung, Kaiser-Wilhelm-Platz 1, Mülheim an der Ruhr 45470, Germany; ‡ Inorganic Chemistry Laboratory, National and Kapodistrian University of Athens, Panepistimiopolis, Zografou 15771, Greece

## Abstract

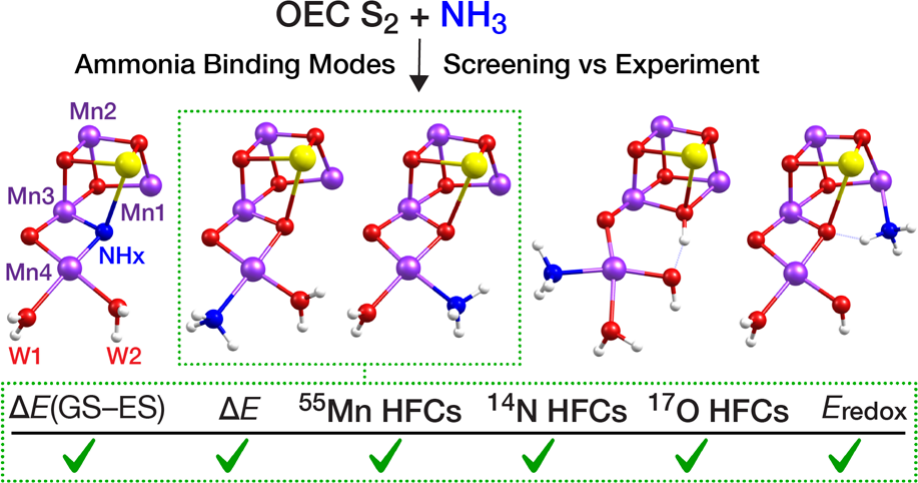

The identity and insertion pathway of the substrate oxygen atoms that are coupled to dioxygen by the oxygen-evolving complex (OEC) remains a central question toward understanding Nature’s water oxidation mechanism. In several studies, ammonia has been used as a small “water analogue” to elucidate the pathway of substrate access to the OEC and to aid in determining which of the oxygen ligands of the tetramanganese cluster are substrates for O–O bond formation. On the basis of structural and spectroscopic investigations, five first-sphere binding modes of ammonia have been suggested, involving either substitution of an existing H_2_O/OH^–^/O^2–^ group or addition as an extra ligand to a metal ion of the Mn_4_CaO_5_ cluster. Some of these modes, specifically the ones involving substitution, have already been subject to spectroscopy-oriented quantum chemical investigations, whereas more recent suggestions that postulate the addition of ammonia have not been examined so far with quantum chemistry for their agreement with spectroscopic data. Herein, we use a common structural framework and theoretical methodology to evaluate structural models of the OEC that represent all proposed modes of first-sphere ammonia interaction with the OEC in its S_2_ state. Criteria include energetic, magnetic, kinetic, and spectroscopic properties compared against available experimental EPR, ENDOR, ESEEM, and EDNMR data. Our results show that models featuring ammonia replacing one of the two terminal water ligands on Mn4 align best with experimental data, while they definitively exclude substitution of a bridging μ-oxo ligand as well as incorporation of ammonia as a sixth ligand on Mn1 or Mn4.

## 1 Introduction

**Figure 1:**
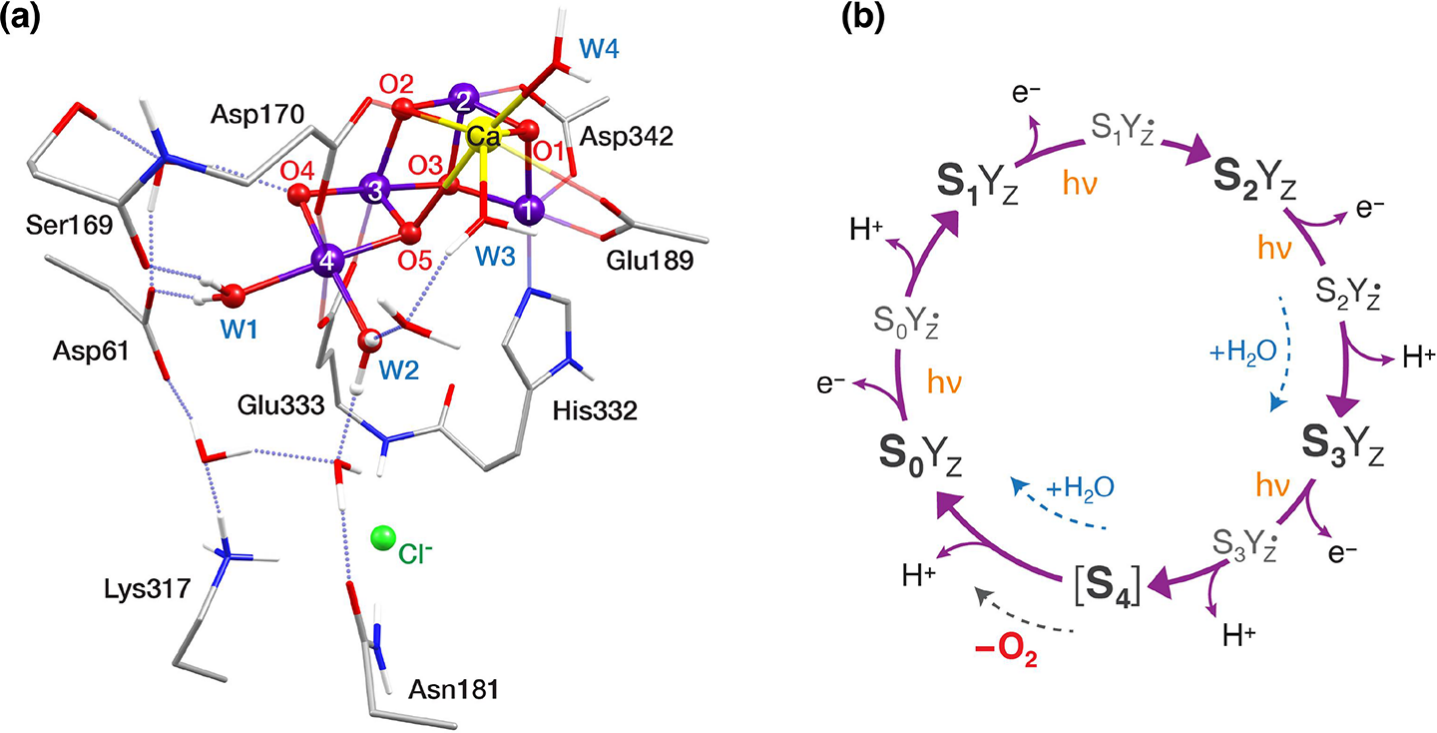
(a) Structure of the OEC in the S_2_ state with selected first and second sphere residues, showing important hydrogen bonding interactions. Mn ions are shown in purple, Ca in yellow, O in red, N in blue, C in gray, and H in white. H atoms attached to C are omitted for clarity. (b) S-state cycle of water oxidation by the OEC.

The oxygen-evolving complex (OEC) of photosystem II (PSII) catalyzes the four-electron oxidation of two substrate water molecules to molecular oxygen.^1,2^ The inorganic core of the OEC is a Mn_4_CaO_5_ cluster (Figure 1a) whose dark-stable state (S_1_) can be described as a near-cuboidal Mn_3_CaO_4_ unit connected to the fourth manganese center (Mn4) via two bridging oxygen atoms, O4 and O5.^3,4^ The complex progresses through a cycle of five states denoted as S_0_–S_4_, where the subscripts represent the number of accumulated oxidative equivalents (Figure 1b).^5−9^ Starting from the most reduced state S_0_,^10^ three sequential Mn(III) → Mn(IV) oxidation events lead to the S_3_ state,^11−19^ and dioxygen is evolved during the S_3_ → [S_4_] → S_0_ transition.^20−22^ Meanwhile, the sequential removal of protons and electrons during the S-state cycle serves to maintain the redox potential of the cluster, effectively reducing the overpotential associated with water oxidation.^23,24^

Two substrate water molecules need to be inserted into the cluster in each catalytic cycle and both are already bound to or near the OEC already in the S_2_ state.^25−27^ The identity of these water molecules as well as of the substrate oxygen atoms involved in the O–O bond formation are among the most significant points to be resolved about the water oxidation mechanism.^6,8,28−30^ These questions remain open because of the difficulty in assigning precise roles to the water channels surrounding the OEC,^30−39^ and the near impossibility of monitoring individual water molecules along the S-state transitions. Instead, the interaction of small molecules such as methanol and ammonia with the Mn_4_CaO_5_ cluster has been studied extensively to provide relevant insights into water delivery, water uptake, and the kinetics of O–O bond formation.^40−51^

This work focuses on ammonia binding on the S_2_ state of the OEC as a substrate analogue. Previous research concludes that ammonia shows at least two different binding modes to the OEC in the S_2_ state,^52,53^ denoted as “primary” and “secondary”, which differ in their spectroscopic properties and reactivity. When ammonia remains on the “secondary” binding site, the electron paramagnetic resonance (EPR) signals between ammonia-treated and untreated PSII samples in the S_2_ state are identical, which indicates noncovalent interaction of ammonia with the Mn_4_CaO_5_ cluster. This binding is competitive with chloride, pH-dependent, and it inhibits oxygen evolution.^52−55^ By contrast, ammonia-treated samples illuminated at 200 K and subsequently annealed above 250 K exhibit altered EPR signals, suggesting direct binding of ammonia to the Mn tetramer at higher temperatures.^56^ In this study, we focus on this “primary” ammonia binding mode. This binding is chloride- and pH-independent,^52,53,57,58^ and the OEC maintains its activity, albeit at a reduced rate of oxygen evolution.^59,60^ Ammonia is released either before the transition to the S_3_ state or between the S_3_ and S_1_ of the succeeding cycle.^60^ Thus, depending on when ammonia release takes place, identification of the specific ammonia binding site(s) has direct implications for substrate water exchange in the S_2_ state, or for the elusive O–O bond formation mechanism.

The “primary” ammonia binding mode has been extensively studied with a range of spectroscopic techniques. Both the S_2_ and ammonia-bound S_2_-state EPR signals arise from an effective ground state spin *S*_GS_ = 1/2 with oxidation states of the four Mn ions Mn(III)Mn(IV)_3_.^17,18,61−64^ The untreated S_2_ also exhibits signals with *g* ∼ 4 and *g* ∼ 5 attributed to high-spin forms, which are not observed upon ammonia binding.^58,65^ Ammonia perturbs the hyperfine interactions between the four ^55^Mn (*I* = 5/2) nuclei and the electron spin, which gives rise to the multiline structure of the *g* ∼ 2 EPR signal. The covalent binding of ammonia to a Mn ion has also been demonstrated by the appearance of a significant ^14^N (*I* = 1) nucleus isotropic hyperfine interaction. Based on the high asymmetry (*η* = 0.4–0.6) of its nuclear quadrupole interaction (NQI),^45,46,58^ ammonia has been suggested to coordinate either as an amido bridge between two metal ions^58^ or as a terminal ligand on Mn4 on the W1 site in the hydrogen-bonding distance from the negatively charged Asp61 residue.^43,45^ Both of these hypotheses have been supported by low-frequency FTIR spectroscopy, which revealed the loss of a vibrational mode^66^ assigned to a Mn–O–Mn or Mn–O–Ca group.^67^ Despite a significant body of experimental and computational work, the relevant literature still contains conflicting models and hypotheses that have been advanced to explain experimental observations.

**Figure 2:**
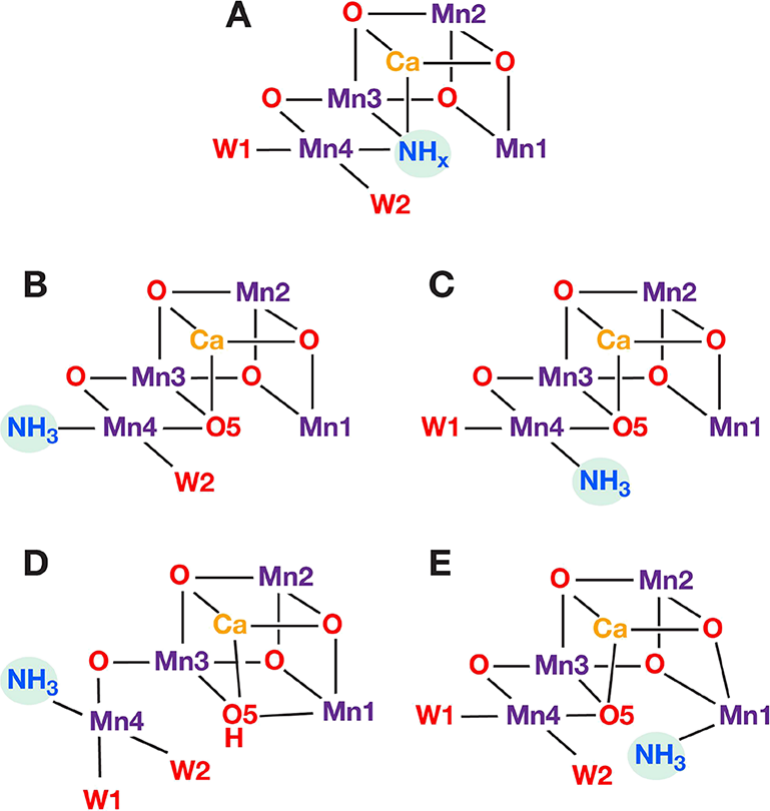
Schematic depiction of direct ammonia interaction modes with the Mn_4_CaO_5_ cluster of the OEC: Binding modes A–D involve ammonia coordination on the Mn4 ion, replacing O5 in A, W1 in B and D and W2 in C, while mode E involves ammonia coordination on Mn1.

**Figure 3:**
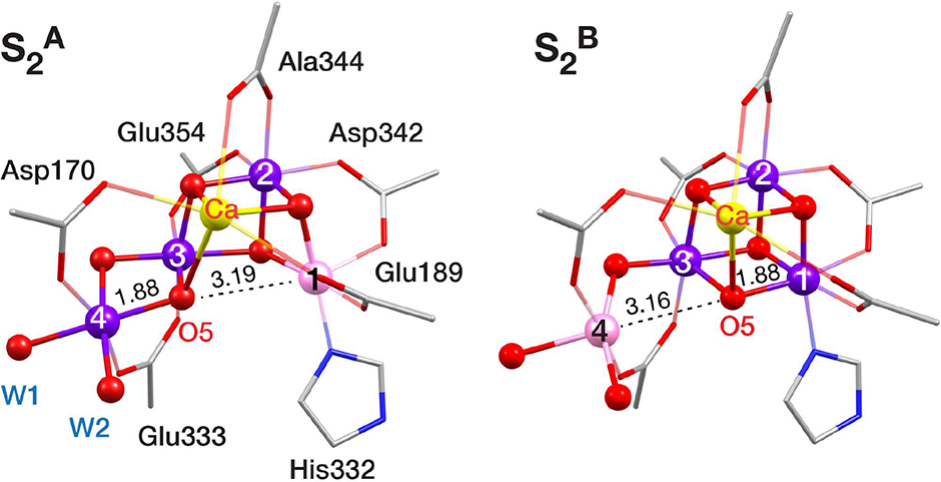
Open-cubane (left) and closed-cubane (right) conformations of the Mn_4_CaO_5_ cluster in the S_2_-state of the OEC cycle, with the Mn1–O5 and Mn4–O5 distances of the optimized structures.

Five basic types of direct ammonia coordination in the S_2_ state of the OEC have been put forward (Figure 2) and have been used as a basis to explain experimental observations. Based on magnetic spectroscopy studies, Britt et al.^58^ first suggested that ammonia substitutes the O5 bridge (Figure 2, mode A). Later, spectroscopic data combined with quantum chemistry calculations reported by Perez Navarro at al.^43^ and by Lohmiller et al.,^45^ as well as by Schraut and Kaupp in the most extensive computational work available to date,^68^ demonstrated that the substitution of the terminal water W1 ligand on Mn4 (mode B) is in better agreement with ^14^N and ^17^O hyperfine coupling constants (HFCs) than O5 substitution.^45,68,69^ More recent crystallographic data by Young et al.^70^ were interpreted as W2 substitution (mode C). Besides, the possibility of ammonia binding as an *additional* ligand on the OEC cluster, without removing any of the ligands, was recently considered. Based on QM/MM calculations, Askerka et al.^71^ suggested that ammonia interacts with a high-spin form of the S_2_ state,^71^ denoted as “closed-cubane” conformation,^72^ in which O5 coordinates on Mn1 whereas Mn4 has an open coordination site (Figure 3). They described a “carousel” mechanism of ammonia binding to Mn4 as W1 and W2 move toward O5 (mode D). In a later computational study, Pushkar et al.^73^ proposed ammonia binding to the open coordination site of Mn1 in the open-cubane S_2_ state conformation (mode E). Recently Dau and co-workers^69,74^ suggested that two or even three ammonia-bound species might coexist in equilibrium in the S_2_ state; therefore, multiple of the above binding modes might be operative.

The spectroscopic parameters of the more recently proposed binding modes C, D, and E, have not yet been computed and, hence, their fitness remains unknown. Herein, we compare the suggested ammonia binding motifs against available experimental data using a common computational framework. Large computational models representing variations of all five ammonia binding modes were constructed and screened, initially according to their effective ground spin states and relative energies, and subsequently evaluated against electron–nuclear double resonance (ENDOR), electron spin echo envelope modulation (ESEEM), and electron–electron double-resonance–detected NMR (EDNMR) spectroscopic data as well as against experimentally determined electron affinities. Our results favor terminal ligand W1 or W2 substitution by ammonia (modes B and C) and disfavor binding modes A, D, and E. The most favored models are energetically close, indicating that they could coexist.

## 2 Methodology

### 2.1 Construction of OEC Models

**Figure 4:**
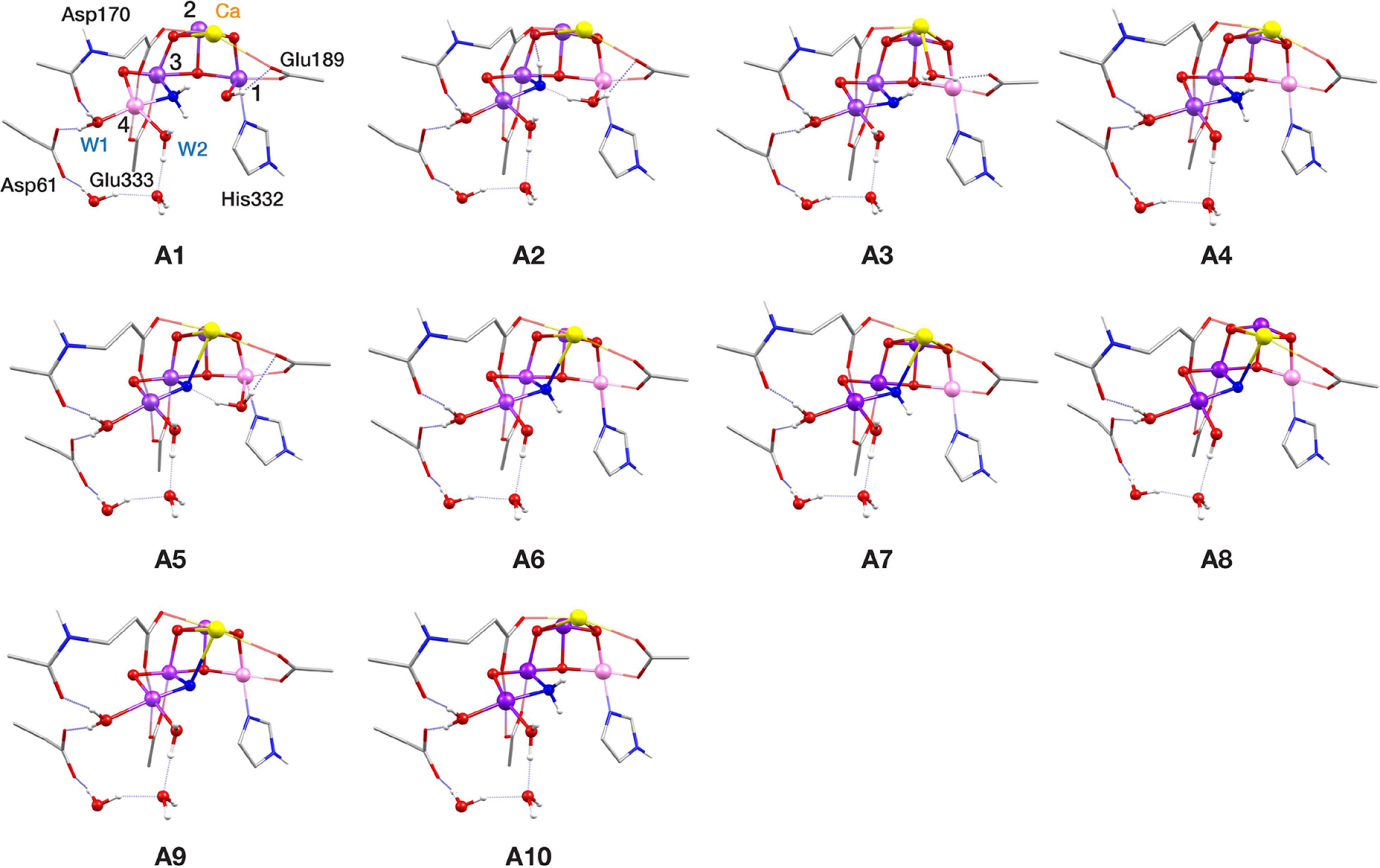
Core structures of S_2_–NH_3_ models with the O5 substitution binding pattern (mode A). Mn(III) ions are indicated in pink, Mn(IV) in dark purple, Ca in yellow, N in blue and O in red. Note that only a small part of the complete computational models is shown (see Figure S1 for a complete model), in order to more clearly depict the major structural features.

**Figure 5:**
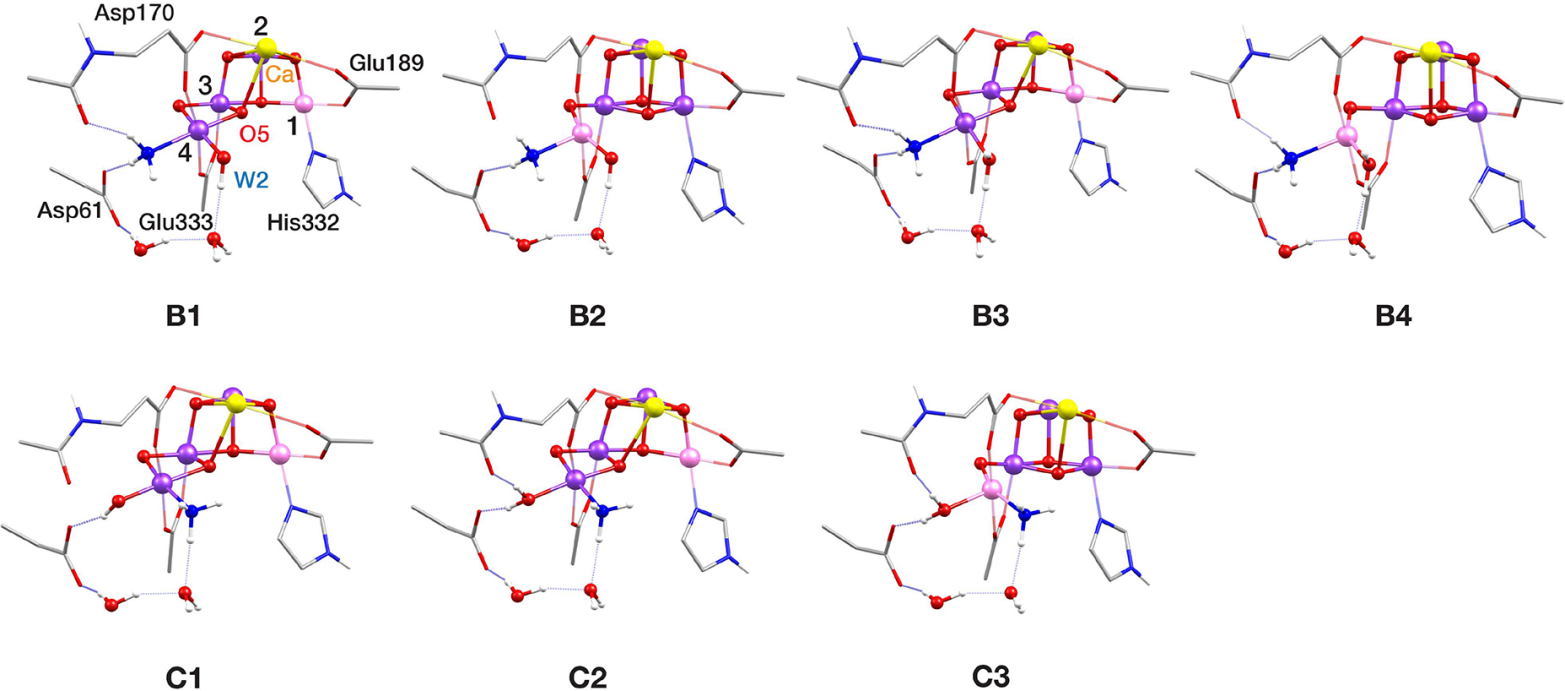
Core structures of S_2_–NH_3_ models with W1 and W2 substitution binding patterns (modes B and C, respectively). Mn(III) ions are indicated in pink, Mn(IV) in dark purple, Ca in yellow, N in blue and O in red.

**Figure 6:**
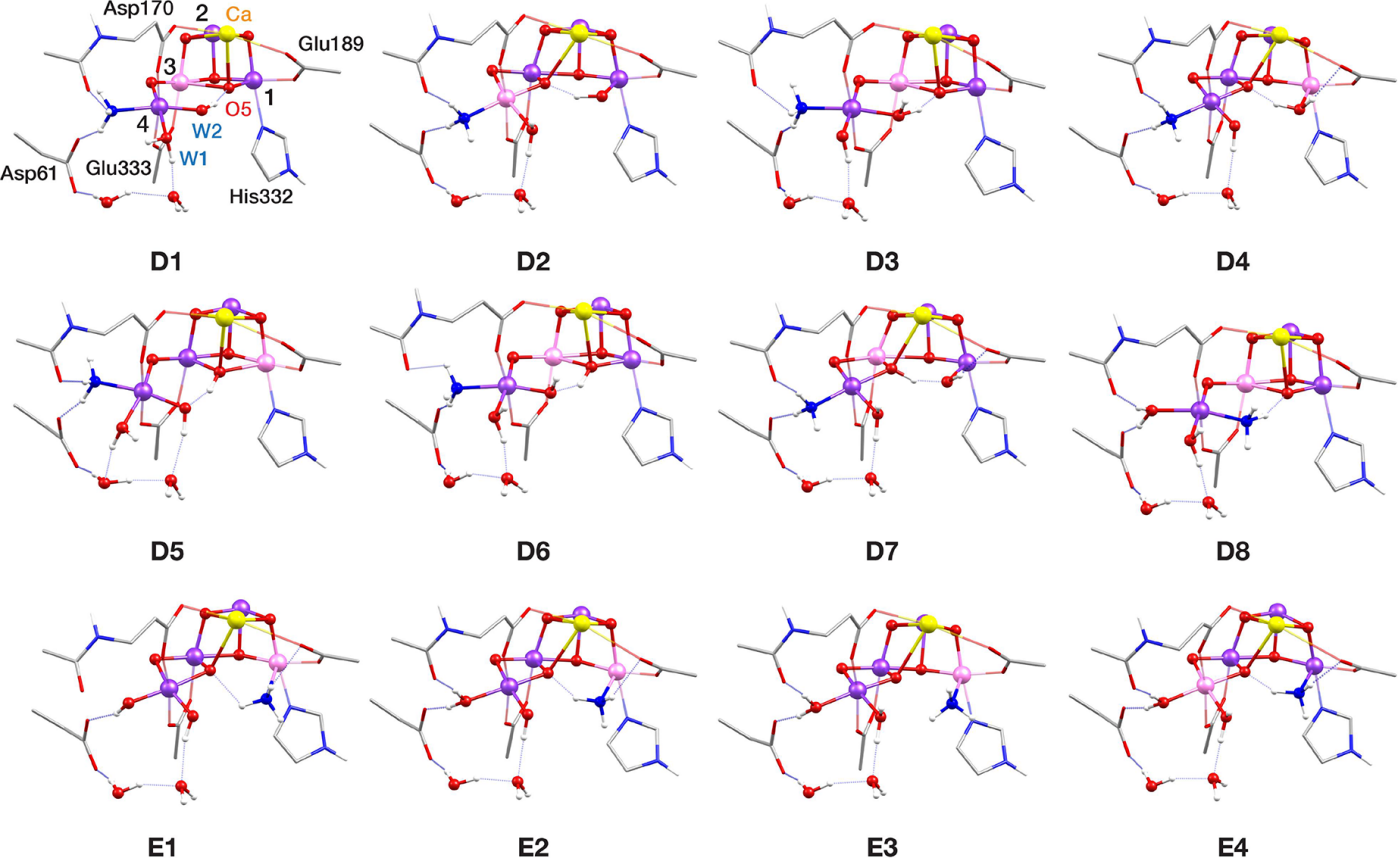
Core structures of S_2_–NH_3_ models with Mn4 and Mn1 addition binding patterns (modes D and E, respectively). Mn(III) ions are indicated in pink, Mn(IV) in dark purple, Ca in yellow, N in blue and O in red.

Models of the OEC in the native S_2_ as well as ammonia-bound S_2_-state with various substitution patterns consist of ca. 350 atoms and were constructed starting from the highest-resolution (1.85 Å) available X-ray diffraction model of PSII (PDB ID 5B66, monomer A) reported by Tanaka et al.^75^ The models include the inorganic core Mn_4_CaO_5_, first coordination sphere amino acids Asp170, Glu189, His332, Glu333, Asp342, Ala344, and CP43-Glu354, and terminal water molecules W1–W4. Moreover, the second coordination sphere amino acids Asp61, Tyr161, Gln165, Ser169, Asn181, Val185, Phe186, His190, Asn298, Lys317, His337, Leu343, and CP43-Arg357, one chloride ion (Cl^–^), and 13 more crystallographic water molecules are included. The cluster model of the S_2_ state is shown in Figure S1 and the Cartesian coordinates of all models are provided as SI material. Starting from the geometry-optimized S_2_ state models, ammonia-bound S_2_ state models (S_2_–NH_3_) were constructed, inspired from literature suggestions about ammonia binding on the first coordination sphere of the OEC. The cores of the 29 models are shown in Figures 4–6.

### 2.2 Screening Criteria

Evaluation of the S_2_–NH_3_ models is primarily based on the predicted ground state spin (*S*_GS_ = 1/2), given that the ammonia-treated S_2_ state exhibits a *g* ∼ 2 multiline EPR signal attributed to a doublet ground spin state. We herein report on the models with predicted *S*_GS_ = 1/2 for which the calculated metal (^55^Mn) and ligand (^14^N and ^17^O) HFCs are in best agreement with those determined experimentally by ^55^Mn ENDOR, ^14^N ESEEM, and ^17^O EDNMR spectroscopy. In addition to spectroscopy-based evaluation, the relative energies of the different models were considered as a criterion. Thus, the calculated ground state spin and relative energies are considered as the most important criteria to distinguish between the various models. The first magnetically excited state is estimated to be ∼30 cm^–1^ higher,^76,77^ which is also used here as one of the criteria for model discrimination. It is worth noting that structural parameters of the S_2_–NH_3_ models were not used as a criterion, due to the limited reliability of currently available experimental data in capturing the subtle differences anticipated between S_2_–NH_3_ and S_2_.^70,78^ Besides, the only available extended X-ray absorption fine structure (EXAFS) data^79^ are known to be compromised by radiation damage^68^ or contain over-reduced intermediates.^80^ Furthermore, it has been experimentally established that ammonia binding slows down the decay of the S_2_ to S_1_ state.^81^ Thus, we also employed the electron affinity (EA) of the S_2_–NH_3_ models relative to the S_2_ state models as a screening criterion.

### 2.3 Computational Details

All calculations were performed with ORCA 4.2.^82^ Geometry optimizations were performed in the respective high-spin states using the BP86^83,84^ density functional. In all calculations, relativistic effects were considered using the zeroth-order regular approximation (ZORA).^85−87^ Specially adapted segmented all-electron relativistically recontracted^88^ basis sets were used, ZORA-TZVP for Mn, O, and N atoms and ZORA-SVP for C and H atoms. The resolution of identity approximation (RI) along with decontracted auxiliary SARC/J Coulomb fitting basis sets was employed in order to decrease computational time. Sufficiently dense integration grids (Grid4 in ORCA convention) and tight self-consistent field (TightSCF) convergence settings were applied. In addition, the conductor-like polarizable continuum model (C-PCM)^89^ with a dielectric constant of 6.0 was used in all calculations.

Magnetic properties were calculated by the broken symmetry-DFT (BS-DFT) approach using the hybrid meta-GGA TPSSh^90^ functional with the RI approximation to the Coulomb exchange and the chain-of-spheres approximation to exact exchange (RIJCOSX)^91,92^ and with increased integration grids (Grid5 and GridX7 in ORCA convention). The ZORA-def2-TZVP(-f) basis sets^88,93^ were used for Mn, O, and N atoms and ZORA-def2-SVP for C and H atoms. Starting from the high-spin determinant of each structure, seven BS determinants were created by inverting local spins of Mn ions. The calculated energies of the BS determinants were used to determine the pairwise exchange coupling constants, *J*_*ij*_, using singular value decomposition and based on the isotropic Heisenberg Hamiltonian
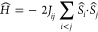


The calculated *J*_*ij*_ values were subsequently used to diagonalize the full Heisenberg Hamiltonian to extract the complete spin ladder and spin projection coefficients. This methodology has been used successfully in a series of previous works.^15,18,72,94−100^

The calculation of hyperfine coupling tensors and nuclear quadrupole tensors was performed on the lowest-energy BS determinant of each model using the TPSSh functional. For the calculation of ^55^Mn, ^14^N, and ^17^O hyperfine coupling tensors and nuclear quadrupole tensors, basis sets were modified with fully decontracted s-functions with three additional steep primitives with exponents 2.5, 6.25, and 15.625 added to the core.^101^ Locally dense radial grids were used for Mn, N, and O atoms (integration accuracy of 11 for Mn and 9 for N and O in ORCA convention). “Picture change” effects that originate from the use of the scalar relativistic Hamiltonian were also included and the complete mean-field approach was used for the spin–orbit coupling operator. Previously reported spin projection techniques were used to transform the results into on-site hyperfine coupling constants.^96,102^ Scaling of DFT-derived values by a factor of 1.78 was used specifically for comparing the computed ^55^Mn hyperfine coupling constants with experimental results.^45,103^ The accuracy of the applied methodology has been quantified in previous benchmark studies on dinuclear Mn complexes.^96,102,103^

## 3 Results

### 3.1 Overview of the Models

To evaluate the different ammonia binding modes, we constructed and optimized large (ca. 350 atoms) cluster models representing several variants of each ammonia-binding mode described in Figure 2. Among the optimized structures, we selected 29 models which describe the full spectrum of possibilities discussed in the literature and calculated their magnetic properties, relative energies, and reduction potentials. For the construction of the different models, we varied the protonation states of W1, W2, and O5 (Figure 1a), and considered the possibility of valence and conformational isomerism, including orientational Jahn–Teller (JT) isomerism. We constructed models with different total numbers of protons, first because the protonation states of the terminal W1/W2 ligands even in the untreated S_2_ state are still debated,^18,48,104,105^ and second because the presence of ammonia or ammonium ions might be changing the protonation state of the OEC. Moreover, we examined models in which the ligand replaced by ammonia either has remained in the cluster as an aquo/hydroxo Mn1 ligand or has left the cluster, i.e. completely removed from the model.

It is worth noting at this point that in the rest of the text, we use the terms “open-” and “closed-” cubane to describe the conformation of the OEC cluster merely in terms of connectivity (Figure 3). Considering that these terms have been previously connected to the idea of valence isomerism in the S_2_ state,^18,72,106^ we clarify that herein we do not associate them with a specific valence distribution in the NH_3_-bound models. The core structures of all geometrically optimized models are presented in Figures 4–6, where Mn(III) ions are shown in pink and Mn(IV) ions in dark purple. In Table S1, the most important structural parameters of all S_2_–NH_3_ models are compared to models of the S_2_ state with W1 in the aquo form and W2 in the hydroxo and aquo form, denoted **S**_**2**_ and **S**_**2**_^**H**^, respectively. Calculated spin populations are listed in Table S2.

In the presentation of the models, we begin with the hypothesis that ammonia substitutes the O5 bridging oxo ligand (binding mode A in Figure 2). Models **A1**–**A10** in Figure 4 resemble intermediates of a mechanism for ammonia binding proposed by Pokhrel and Brudvig.^107^ In the described mechanism, ammonia interacts with the closed-cubane form of the S_2_ state replacing O5, which was suggested to coordinate to the Ca^2+^ ion protonated in the aquo or hydroxo form. During geometry optimizations, O5 leaves the Ca^2+^ ion and binds to the Mn1(III) open coordination site, giving models **A1**, **A2,** and **A5**, whereas it remains on Ca^2+^ in model **A3**. In models **A6**–**A10** the O5 was not included in the starting structures before optimization. The proposed mechanism leads to the formation of an open-cubane structure with valence distribution [III,IV,IV,IV]. Models **A2**–**A10** have the same valence distribution, whereas **A1** is [IV,IV,IV,III]. We note that the corresponding closed-cubane structures were also optimized, but since they are energetically unfavorable and have high-spin ground states (in line also with a previous report^68^), they were not investigated further. While the substitution of the O5 μ-oxo bridge by amido (NH_2_^–^) and imido (NH^2–^) bridges has been previously examined,^68^ substitution by a nitrido bridge (N^3–^), which was suggested^107^ as a more plausible scenario due to the absence of large proton hyperfines^43,51^ has not been studied yet using quantum chemistry. With models **A1**–**A10**, which represent different variants of the O5 bridge substituted by NH_2_^–^ (**A4** and **A10**), NH^2–^ (**A2**, **A3**, **A6,** and **A7**), and N^3–^ (**A5**, **A8,** and **A9**), we revisit and elaborate on the proposed hypothesis under a common framework.

Next, we examine the case of terminal W ligand substitution by ammonia, and the corresponding models are shown in Figure 5. Models **B1**–**B4** derive from the substitution of W1 (mode B). In **B1** and **B2**, W2 is in the hydroxo form, whereas in **B3** and **B4**, it is in the aquo form. **B1** and **B3** are in the open-cubane conformation and have the same valence distribution [III,IV,IV,IV], whereas **B2** and **B4** are closed-cubane with valence distribution [IV,IV,IV,III]. Likewise, ammonia-binding pattern C, where ammonia replaces W2, is represented by models with varying W1 protonation states and locations of the lone Mn(III) ion of the cluster. However, when W1 is in the hydroxo form, only the open-cubane isomer with valence distribution [III,IV,IV,IV] (model **C1**) could be located, since a local minimum of the respective closed-cubane valence isomer [IV,IV,IV,III] was not found, presumably because it is unfavorable for the strong OH^–^ (W1) ligand to be on the Mn4(III) JT elongation axis. In accordance with the previous computational studies,^45,68,69^ terminal substitution ammonia-binding patterns B and C induce minimal structural changes on the S_2_ state (Table S1).

Binding modes D and E (Figure 6) represent ammonia binding as an additional terminal ligand on Mn4 and Mn1, respectively, without exchange with a W ligand, which means that both terminal W ligands and O5 remain coordinated. Models **D1**–**D7** derive from ammonia binding as a sixth ligand on Mn4 at the former W1 position, whereas in **D8** it binds at the open coordination site of Mn4 in the closed-cubane conformation of the cluster. Models **D1**–**D4** are isomers and models **D5**–**D8** have an additional proton. In **D1**, **D3**, **D5,** and **D6**, ammonia binds in the closed-cubane S_2_ conformation, and the positions of W1 and W2 are shifted toward O5, which results in an octahedral Mn4 coordination sphere. The unique Mn(III) ion in the cluster is Mn3 and it has a pseudo-JT elongation axis along the Mn3–O5 direction, except model **D5** which is the only model in this study that adopts the closed-cubane conformation with valence distribution [III,IV,IV,IV]. In **D5** the pseudo-JT elongation axis of Mn1(III) is along the Mn1–O5 bond. This model corresponds to the structure proposed in the QM/MM study by Askerka et al.^71^ to represent the ammonia-bound S_2_ state. Inspired by proposed scenarios of open- and closed-cubane interconversion in the S_3_ and S_4_ states of the OEC,^108−113^ we constructed and optimized **D2**, **D4,** and **D7**, as the open-cubane isomers of **D1**, **D3**, and **D5**–**D6**, respectively. Their valence distributions vary, with **D2** being [IV,IV,IV,III], **D4** [III,IV,IV,IV], and **D7** [IV,IV,III,IV]. In **D2**, W1 is in the aquo form and lies along the Mn4(III) pseudo-JT elongation axis, whereas in **D4** W1 is a hydroxo and the Mn1 coordinating terminal W is protonated instead and lies along the Mn1(III) pseudo-JT elongation axis. Therefore, models **D1**–**D8** exhaustively cover the range of possibilities for ammonia addition on Mn4 in the S_2_ state.

The fifth ammonia-binding pattern, E, is coordination as a sixth ligand on Mn1 (Figure 6). Pushkar et al.^73^ considered a deprotonated S_2_ state as they investigated the reactivity of the OEC at increased pH. In our investigation, we examined different protonation states of the terminal Mn4 W ligands. Model **E1**, having both W1 and W2 in the hydroxo form, corresponds directly to the proposed structure.^73^ In **E2** W1 is protonated in the aquo form and in **E3** and **E4** both W1 and W2 are in the aquo form. In **E1**–**E3** the valence distribution among the four Mn ions of the cluster is [III,IV,IV,IV]. The pseudo-JT elongation axis of Mn1(III) is along the Mn1–NH_3_ vector in **E1** and **E3**, whereas in **E2** it is along the Mn1–N_His332_ vector. Models **E3** and **E4** are valence isomers, with **E4** having [IV,IV,IV,III] valence distribution. The axial elongation of Mn4(III) is along the Asp170-Mn4-Glu333 direction.

To summarize, 29 unique S_2_–NH_3_ models were optimized, and in the next sections, they are systematically examined against available experimental data on ammonia-treated S_2_ samples.

### 3.2 Spin States

The calculated pairwise exchange coupling constants as well as the energy differences between the two lowest spin states of each model are shown in Table 1. The valence distributions in the optimized models are shown in Figures 4–6, where Mn(IV) is shown in dark purple and Mn(III) is in pink, and spin populations are given in Table S2. We observe that each one of the 14 models with a predicted *S*_GS_ = 1/2 has valence distribution [Mn1, Mn2,Mn3,Mn4] = [III,IV,IV,IV]. Their magnetic coupling topology involves antiferromagnetic coupling between Mn1 and Mn2 (*J*_12_ < 0), ferromagnetic coupling between Mn2 and Mn3 (*J*_23_ > 0), and antiferromagnetic coupling between Mn3 and Mn4 (*J*_34_ < 0), except model **A10**, where weak ferromagnetic interaction is predicted for Mn3 and Mn4. For all models, the lowest energy broken-symmetry determinant is the one where Mn ions have local spins *M*_*s*_ (2,–3/2,–3/2,3/2), with the exception of **D5** which has the lowest energy broken-symmetry determinant (2,–3/2,3/2,–3/2) and exhibits a large ferromagnetic coupling between Mn1 and Mn3. In addition, all models with valence distribution [III,IV,IV,IV] exhibit an effective doublet ground spin state, except **A2**, **A3**, **A6,** and **A7**. In these models, the ferromagnetic interaction between Mn3 and Mn4, presumably enabled by the imido (NH) bridging ligand, results in a high-spin ground state.

**Table 1:** Computed Mn–Mn Exchange Coupling Constants (*J*_*ij*_ in cm^–1^), Ground Spin State (*S*_GS_), and First Excited Spin State (*S*_ES_), Their Energy Separation (Δ*E*_ES_ in cm^–1^), and Energy Difference between the Ground State and the Lowest-Lying Spin Doublet State (Δ*E*_*S*=1/2_ in cm^–1^) for All S_2_–NH_3_ Models

	exchange coupling constants, *J*_*ij*_						spin states			
	*J*_12_	*J*_13_	*J*_14_	*J*_23_	*J*_24_	*J*_34_	*S*_GS_	*S*_ES_	Δ*E*_ES_	Δ*E*_*S*=1/2_
**A1**	18.2	0.1	3.6	–3.7	0.5	–32.3	7/2	5/2	41.0	216.9
**A2**	–42.7	3.9	–1.5	12.5	2.1	3.4	5/2	7/2	27.1	49.4
**A3**	–20.3	2.7	6.6	11.6	1.8	12.6	7/2	5/2	17.4	101.8
**A4**	–17.0	–5.4	3.6	8.5	2.0	–2.0	**1/2**	3/2	8.4	
**A5**	–28.9	0.9	0.4	21.0	–2.0	–43.4	**1/2**	3/2	59.7	
**A6**	–14. 8	0.6	6.8	11. 6	2.2	36.3	7/2	5/2	9.4	214.5
**A7**	–12.0	–2.3	7.3	14.3	2.4	39.3	5/2	7/2	1.1	198.7
**A8**	–21.2	10.8	13.5	28.2	0.8	–45.0	**1/2**	3/2	37.2	
**A9**	–17.3	8.9	9.6	24.5	–1.4	–56.8	**1/2**	3/2	36.3	
**A10**	–11.8	–10.4	5.2	12.7	2.0	2.3	**1/2**	3/2	0.8	
**B1**	–17.4	4.4	1.3	19.6	1.9	–10.6	**1/2**	3/2	17. 8	
**B2**	29.3	16.5	13.9	27.5	0.9	–6.9	13/2	11/2	14.2	355.5
**B3**	–14.8	2.7	2.4	20.9	1.7	–9.2	**1/2**	3/2	16.0	
**B4**	33.0	10.7	5.0	31.9	1.9	–2.4	13/2	11/2	16.2	346.9
**C1**	–17.1	6.2	1.1	20.5	0.7	–15.8	**1/2**	3/2	20.4	
**C2**	–15.4	0.2	2.2	17.9	1.7	–10.5	**1/2**	3/2	19.8	
**C3**	32.9	10.4	5.6	29.5	1.5	–12.3	5/2	7/2	4.1	305.4
**D1**	26.4	–7.0	9.3	–27.4	–0.2	–8.3	5/2	3/2	82.1	149.8
**D2**	16.5	–5.5	2.2	8.8	–0.1	–34.1	7/2	5/2	1.6	117.9
**D3**	24.9	–9.2	1.3	–30.1	0.4	–22.7	5/2	3/2	89.6	181.4
**D4**	–42.3	–1.3	–0.5	19.0	1.3	–14.6	**1/2**	3/2	37.7	
**D5**	–31.9	33.7	2.6	28.2	1.4	–21.7	**1/2**	3/2	28.7	
**D6**	28.2	16.6	8.4	–25.7	–0.2	–10.9	5/2	7/2	21.8	110.2
**D7**	18.9	–12.8	1.4	32.8	1.3	–25.8	5/2	7/2	6.1	36.1
**D8**	27.0	1.3	1.4	–24.8	–1.3	–39.2	5/2	3/2	102.3	222.9
**E1**	–34.14	4.49	–0.16	18.74	0.93	–33.13	**1/2**	3/2	58.1	
**E2**	–37.9	–2.0	0.3	17.2	1.8	–19.8	**1/2**	3/2	45.6	
**E3**	–33.7	–1.2	–0.6	16.3	1.6	–19.1	**1/2**	3/2	42.4	
**E4**	5.9	–3.4	0.1	16.8	0.4	–92.3	5/2	3/2	27.4	44.1
**S**_**2**_	–17.3	1.8	1.6	17.0	2.2	–15.6	**1/2**	3/2	25.7	
**S**_**2**_^**H**^	–15.0	0.1	2.3	18.9	2.0	–12.4	**1/2**	3/2	21.3	

Models with predicted ground states with *S*_GS_ > 1/2 are considered inconsistent with the experiment since ammonia-treated samples exhibit a *g* ∼ 2 EPR signal attributed to a doublet ground spin state. For all models with predicted *S*_GS_ = 1/2 the first excited spin state has *S*_ES_ = 3/2. EPR studies support a first excited spin state on the order of ∼ 30 cm^–1^ for the ammonia-treated and ∼ 36 cm^–1^ for the untreated S_2_ state.^76,77^ As observed in Table 1, the largest deviations from this value are calculated for models **A4**, **A5**, **A10,** and **E1**; for **A4** and **A10** the computed energy difference is less than 10 cm^–1^, whereas for **A5** and **E1** it is almost 60 cm^–1^. For all other doublet S_2_–NH_3_ models, the energy difference between the two lowest states of the spin ladder is within 16–46 cm^–1^. Notably, for all models with a predicted ground state with *S*_GS_ > 1/2, the energy difference (Δ*E*_*S*=1/2_) between the ground state and the lowest-lying spin doublet state is larger than 36 cm^–1^ (Table 1), showing there is little uncertainty regarding the assignment of ground spin state, in view of the known performance of the applied computational protocols.^15,18,72,94−100,103,114^ In the rest of this work, we will compute the EPR parameters and discuss further the 14 models with predicted *S*_GS_ = 1/2, i.e., **A4**, **A5**, **A8**, **A9**, **A10**, **B1**, **B3**, **C1**, **C2**, **D4**, **D5**, **E1**, **E2**, and **E3**.

### 3.3 Relative Energies

A limitation in evaluating the models in terms of energetics is that not all of them are isomers, as they have different total numbers of H and O atoms. Therefore, we define four subsets of isomer structures, namely, 2O–4H, 2O–5H, 3O–6H, and 3O–7H, and we compare the relative energies between the models of each subset. In the above subset labels, 3O means that W1, W2, and O5 ligands still remain in the cluster, whereas 2O means that one of these ligands is removed, after ammonia binding. The label *n*H refers to the total number (*n*) of protons on the ligands W1, W2, and O5, and on the ammonia-derived nitrogen ligand (ammonia, imido, or imino ligands). For example, model **B1** belongs to the 2O–4H subgroup because it only has W2 and O5 and because the total number of protons on ammonia, W2, and O5 is four. It follows that models **A8**, **A10,** and **E1**, which do not belong to any of these subsets, are not included in this comparison.

**Figure 7:**
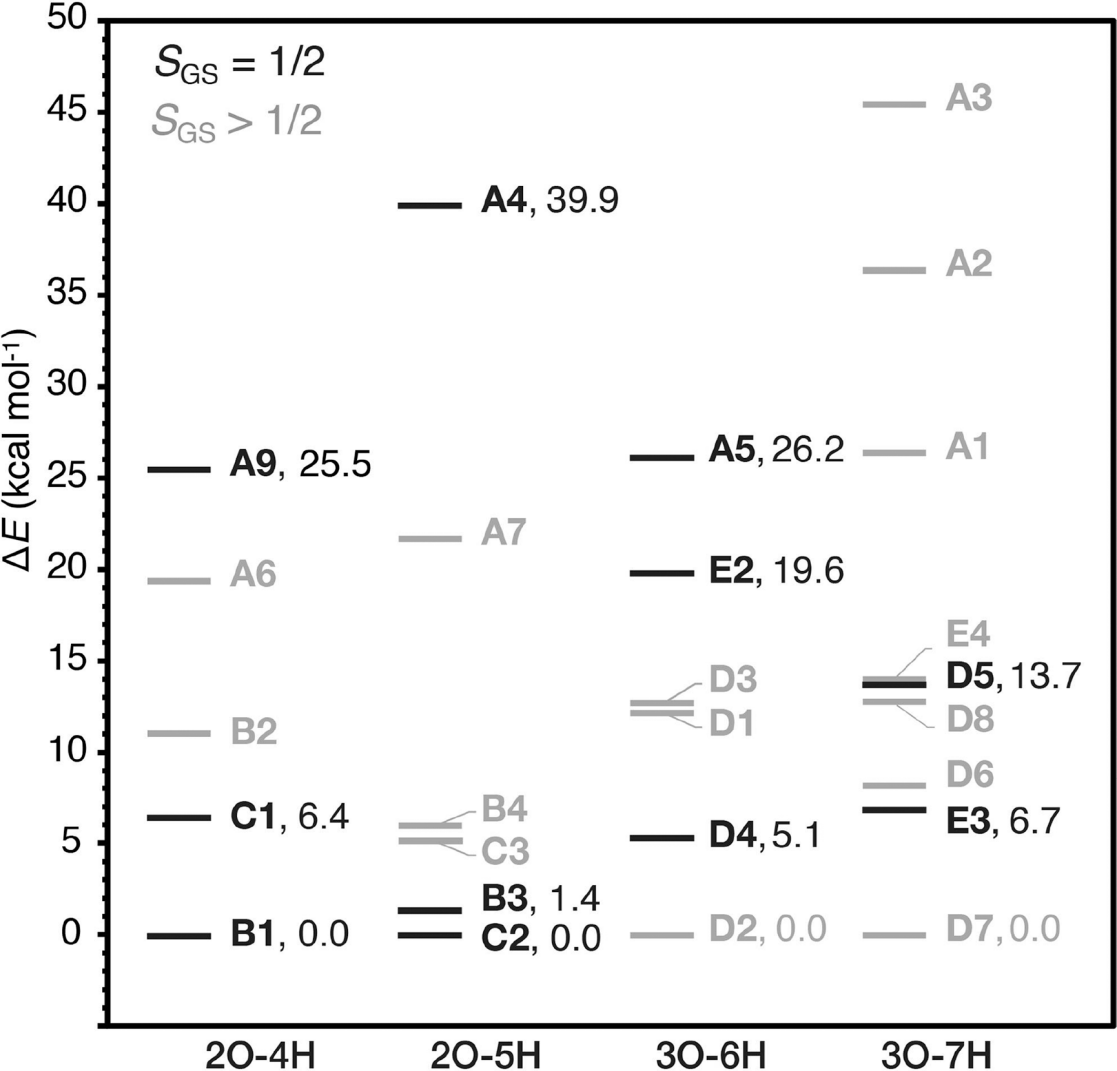
Relative energies of the lowest-energy BS-TPSSh determinants of the S_2_–NH_3_ models divided into four subsets of isomer structures. The relative energies computed with different computational approaches are given in Table S3. Models with doublet ground spin states are indicated in black.

The relative energies among the models that belong to each subset are plotted in Figure 7. The models with predicted *S*_GS_ = 1/2 are shown in red. It can be seen that models in which the O5 bridge is replaced by N (mode A) are strongly energetically unfavorable, being the highest in energy among the models of each subset. In subsets 2O–4H and 2O–5H, terminal water substitution models (modes B and C), are the lowest in energy. Among these models, spin doublet ground state structures **B1**, **B3**, and **C2** are lower than the corresponding high-spin isomers **B2**, **B4,** and **C3**, respectively. For the most favorable models **B1**, **B3**, **C1,** and **C2**, it can be also observed that when the Mn4(IV) terminal W ligand is in the hydroxo form (2O–4H subset), W1 substitution is favored over W2 substitution (**B1** 6.5 kcal mol^–1^ lower than **C1**), whereas when the terminal W ligand is in the aquo form (2O–5H subset), W2 substitution is preferred (**C2** 1.4 kcal mol^–1^ lower than **B3**).

Turning now to the 3O–6H and 3O–7H subsets, the high-spin models **D2** and **D7** are lower in energy than the low-spin isomers, **D4** and **E3**, respectively. This means that even if **D4** or **E3** are found to be consistent with the available spectroscopic data, they would still not be considered energetically favorable. The 3O–6H and 3O–7H plots also reveal that among the models with binding mode D, closed-cubane structures **D1**, **D3** and **D5**, **D6**, and **D8** are higher in energy than the corresponding open-cubane isomers **D2**, **D4**, and **D7**, regardless of the valence distribution among the Mn ions. It is also important to point out that among the valence isomers **D5** and **D6**, the low-spin **D5** is 5.5 kcal mol^–1^ higher than **D6**. In addition, those results show that binding of ammonia as an additional ligand to Mn4 is more favorable energetically over binding to Mn1. Overall, energy-based evaluation of the models suggests **B1** and **C2** as the most energetically favored models, whereas O5 substitution models can be ruled out on energetic grounds.

### 3.4 EPR Parameters

#### 3.4.1 ^55^Mn HFCs

**Figure 8:**
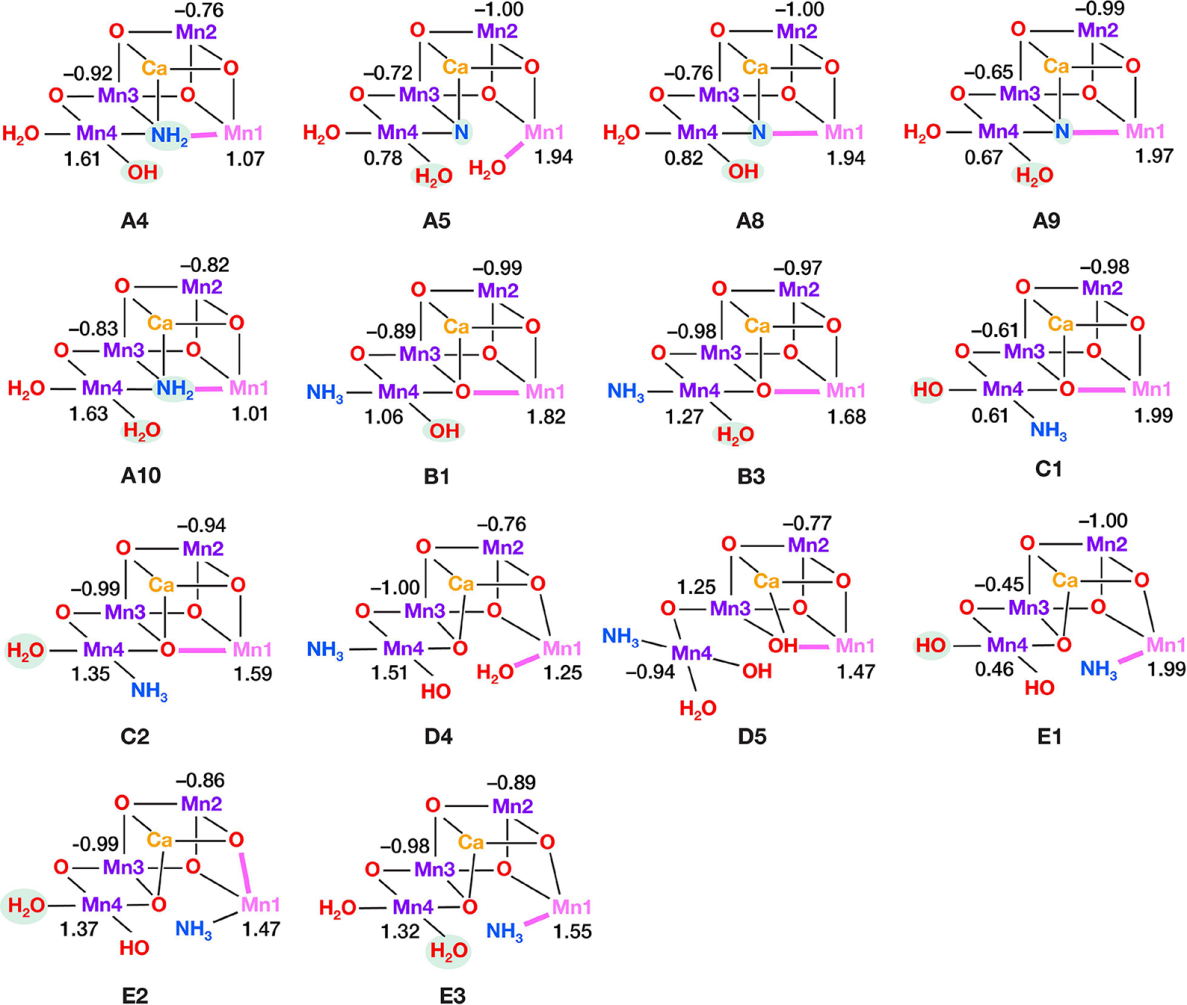
Mn_4_CaO_5_–NH_*x*_ cores of the 14 S_2_–NH_3_ models with predicted doublet ground spin states (*S*_GS_ = 1/2) showing the calculated Mn spin projection coefficients (*ρ*_*i*_). Mn(III) ions are shown in pink and Mn(IV) in purple. Bonds along the pseudo JT axes of Mn(III) ions are shown in pink bold lines.

The calculated ^55^Mn isotropic HFCs for all models featuring *S*_GS_ = 1/2 (Figure 8) are given in Table 2 and compared with ^55^Mn HFCs obtained from ENDOR experiments.^45,51^ Given that the latter fitted effective hyperfine tensors cannot be straightforwardly assigned to specific Mn ions of the tetramanganese cluster, comparison to the experiment is based exclusively on the HFC absolute values. Thus, the computed HFCs are arranged in descending order of |*A*_iso_| magnitude, i.e., *A*_1_ > *A*_2_ > *A*_3_ > *A*_4_, with the corresponding Mn ion indicated in square brackets in Table 2.

**Table 2:** Calculated Effective/Projected and Experimental Isotropic Hyperfine Coupling Constants (|*A*_iso_|, in MHz) for the Mn Ions of the S_2_–NH_3_ Models with *S*_GS_ = 1/2 and of the *S*_2_ State Models, Arranged in the Descending Order, i.e., *A*_1_ > *A*_2_ > *A*_3_ > *A*_4_, with the Corresponding Mn Ion Indicated in Square Brackets, and Ratios *A*_1_/*A*_2_, *A*_2_/*A*_3_, *A*_3_/*A*_4_

	*A*_1_	*A*_2_	*A*_3_	*A*_4_	*A*_1_/*A*_2_	*A*_*2*_/*A*_3_	*A*_3_/*A*_4_
**A4**	448 [4]	216 [3]	189 [2]	188 [1]	2.07	1.14	1.01
**A5**	416 [1]	235 [4]	247 [2]	190 [3]	**1.77**	**0.95**	**1.30**
**A8**	315 [1]	258 [2]	243 [4]	192 [3]	**1.22**	**1.06**	**1.27**
**A9**	339 [1]	250 [2]	201 [4]	172 [3]	**1.35**	**1.25**	**1.16**
**A10**	451 [4]	203 [3]	199 [2]	191 [1]	2.22	1.02	1.04
**B1**	311 [1]	291 [4]	248 [2]	206 [3]	1.07	1.17	1.21
**B3**	349 [4]	292 [1]	243 [3]	237 [2]	**1.19**	**1.20**	**1.03**
**C1**	339 [1]	247 [2]	169 [4]	139 [3]	1.37	1.46	1.21
**C2**	374 [4]	276 [1]	247 [3]	233 [2]	**1.35**	**1.12**	**1.06**
**D4**	409 [4]	329 [1]	227 [3]	183 [2]	1.24	1.45	1.24
**D5**	342 [1]	342 [3]	238 [4]	223 [2]	1.00	1.44	1.07
**E1**	447 [1]	252 [2]	134 [4]	100 [3]	1.78	1.88	1.35
**E2**	386 [4]	339 [1]	232 [3]	202 [2]	1.14	1.46	1.15
**E3**	368 [4]	347 [1]	243 [3]	214 [2]	1.06	1.43	1.13
*exp.*							
*Synechocystis*(51)	313	206	198	153	1.52	1.04	1.29
*Spinach*(51)	318	205	193	160	1.55	1.06	1.21
*T. vestitus*(45)	331	231	225	186	1.43	1.03	1.21
S_2_ state without ammonia:							
**S**_**2**_	313 [4]	302 [1]	246 [2]	210 [3]	1.04	1.23	1.17
**S**_**2**_^**H**^	368 [4]	284 [1]	244 [3]	234 [2]	1.29	1.17	1.04
*exp.*							
*Synechocystis*(51)	307	209	204	190	1.47	1.02	1.07
*Spinach*(51)	310	242	205	194	1.28	1.18	1.06
*T. vestitus*(45)	333	230	227	194	1.45	1.01	1.17

Interestingly, even though the experimental *A*_1_ HFC is usually attributed to Mn1(III) based on its lower valence,^45,51^ calculations show that this is not necessarily the case.^18,45,68,115,116^ As shown in Table 2, the largest |*A*_iso_| value is computed for Mn1 in some models and for Mn4 in others. To explain the origins of this observation, we stress that the effective (spectroscopically observed) hyperfine coupling constant of each ion Mn_*i*_ of the cluster results from its local hyperfine coupling constant (*α*_*i*_) scaled by the contribution of its electronic spin to the effective spin state of the cluster, according to the equation: *A*_i_ = *ρ*_*i*_*α*_*i*_, where *ρ*_*i*_ is the projection coefficient.^96^ This means that the effective HFC of each individual Mn ion is affected by the overall electronic structure of the cluster and specifically by the exchange interactions between all Mn ions. The computed spin projection coefficients for all doublet ground spin state models are shown in Figure 8. It is noted that for all models the signs of the spin projection coefficients of the Mn ions of the cluster are in agreement with the lowest energy BS determinant, i.e., αββα and αβαβ for **D5**. The Mn2 and Mn3 spin projection coefficients (absolute values) range between 0.45 and 1.25, for Mn1 between 1.07 and 1.99, and for Mn4 between 0.46 and 1.63. Thus, in models **A4**, **A10**, **B3**, **C2**, **D4**, **E2**, and **E3**, the large Mn4 spin projection coefficient results in Mn4 |*A*_iso_| larger than Mn1 |*A*_iso_|.

The electronic structure of each Mn ion is reflected on its local hyperfine coupling tensor. The computed local ^55^Mn isotropic *α*_iso_ and anisotropic *α*_aniso_ HFCs for all models are listed in Table S4. It can be seen that octahedral Mn(IV) ions, i.e., Mn2, Mn3, and Mn4, exhibit hyperfine values |*α*_iso_| within 219–301 MHz with small anisotropy |*α*_aniso_| < 49 MHz. Interestingly, Mn4(IV) exhibits the largest |*α*_iso_| in all models, except **D5** for which Mn2(IV) has the largest |*α*_iso_|. Five-coordinated square-pyramidal Mn1(III) ions in structures with open cubane geometry, found in structures **A4**, **B1**, **B3**, **C1** and **C2**, have a calculated |*α*_iso_| near 140 MHz, whereas those of hexa-coordinated octahedral Mn(III) ions, found in structures **A5**, **D4**, **D5**, **E1**, **E2** and **E3**, are within 191–231 MHz. The difference between the magnitude of |*α*_iso_| between a square-pyramidal d^4^ Mn(III) ion and a tetragonally elongated octahedral Mn(III) ion can be associated with the ground state symmetry,^117−119^ i.e., formal ^5^*B*_1*g*_ and ^5^*B*_1_ ground states for the five- and six-coordinated Mn(III) ions, respectively. Besides, Mn(III) ions exhibit large anisotropy of the calculated hyperfine tensors, with |*α*_aniso_| between 141 and 150 MHz. Overall, the calculated local HFCs are determined by the valence and coordination environment of each Mn ion, and differences among the computed observable (projected) HFCs of the proposed models can be mainly attributed to differences between the spin projection factors.

An obvious challenge in comparing the computed HFCs given in Table 2 to ^55^Mn ENDOR-derived values is that experimental isotropic HFCs differ considerably among spinach and cyanobacteria, by as much as 27 MHz. To eliminate this issue, we introduce the ratios *A*_1_/*A*_2_, *A*_2_/*A*_3_, *A*_3_/*A*_4_ as a more appropriate criterion. As shown in Table 2, these ratios are very similar among different organisms, with *A*_1_/*A*_2_ close to 1.5, *A*_2_/*A*_3_ to 1.0, and *A*_3_/*A*_4_ to 1.2. This criterion has the additional advantage of being independent of the scaling factor applied to compare with experimental results (here 1.78, see Computational Details). Examination of the ratios of the calculated HFCs for the S_2_–NH_3_ models shows that **A5**, **A8**, **A9**, **B3,** and **C2** are in the best agreement with ^55^Mn ENDOR data. By contrast, for all models with binding modes D and E, the *A*_2_/*A*_3_ ratio well exceeds the experimental ratio since *A*_2_ is much larger than *A*_3_ for each of these models. Thus, binding modes A, B, and C are more in line with ^55^Mn ENDOR experiments.

#### 3.4.2 ^14^N HFCs

Next, we focus on ligand HFCs and compare our results with those obtained from ESEEM and EDNMR experiments. The calculated ^14^N isotropic HFCs (|*A*_iso_|) and the nuclear quadrupole asymmetry parameter (*η*) for the NH_3_ and His332 ligands for the S_2_–NH_3_ models are presented in Table 3. In addition, the dipolar and rhombicity terms of the calculated ^14^N hyperfine tensors and the NQI terms of both the S_2_–NH_3_ models and the native S_2_ state models are given in Table S5. Regarding the His332 ligand, ESEEM experiments have demonstrated that ammonia binding does not affect the hyperfine coupling tensor of the Mn1 coordinating ^14^N nucleus.^45,46^ Models **D4**, **E1**, **E2**, and **E3** are not consistent with this observation, as their ^14^N His332 |*A*_iso_| values are significantly different from those of the S_2_ state (Table 3), which can be directly attributed to the large perturbation of their Mn1 coordination spheres (Table S1).

**Table 3:** Calculated Effective/Projected ^14^N Isotropic Hyperfine Coupling Constants (|*A*_iso_| in MHz) and Anisotropy of the NQI Tensors in MHz for the Bound NH_*x*_ Nitrogen and the His332 Imino-Nitrogen of the S_2_–NH_3_ Models with *S*_GS_ = 1/2^a^

	NH_*x*_		His332	
	|*A*_iso_|	*η*	|*A*_iso_|	*η*
**A4**	3.1	0.8	3.4	0.9
**A5**	6.4	0.9	2.5	0.6
**A8**	3.6	1.0	5.2	0.5
**A9**	3.6	1.0	5.6	0.6
**A10**	2.3	0.8	3.5	0.8
**B1**	3.0	0.8	5.4	0.7
**B3**	3.5	0.9	5.2	0.8
**C1**	**1.2**	**0.3**	**5.9**	**0.7**
**C2**	**3.7**	**0.3**	**4.9**	**0.8**
**D4**	4.6	0.9	1.0	0.6
**D5**	**2.0**	**0.7**	**3.6**	**0.7**
**E1**	13.7	0.3	1.2	0.5
**E2**	3.2	0.7	10.2	0.2
**E3**	10.6	0.2	1.1	0.6
*exp.*				
*Synechocystis*(46)	2.3	0.4	7.2	0.8
*Spinach*(58)	2.3	0.6		
*T. vestitus*(45)	2.4	0.5	7.2	0.8
S_2_ state without ammonia:				
**S**_**2**_			5.4	0.7
**S**_**2**_^**H**^			5.1	0.8
*Exp.*				
*Synechocystis*(46)			7.2	0.8
*T. vestitus*(45)			7.1	0.8

a Results are compared with parameters fitted from ^14^N ESEEM spectra. The ^14^N isotropic hyperfine coupling constants for the His332 imino-nitrogen of the S_2_ state are also given.

Regarding the calculated ^14^N isotropic HFCs for the NH_*x*_ ligand, the largest deviations from the experiment are observed for **A5**, **E1,** and **E3**. In **E1** and **E3**, ammonia coordinates to Mn1(III) as an axial ligand along its JT elongation axis (Figure 8), leading to a strong interaction between the ^14^N nucleus and the electron spin of the d_*z*^2^_ orbital of Mn1(III). Moreover, the asymmetry of the ^14^N NQI can be considered as a probe of the environment of the inserted ^14^N ligand. As shown in Table 3, ammonia-treated samples of different organisms exhibit a relatively wide range of *η* values, from 0.4 to 0.6. Models **A4**, **A5**, **A8**, **A9**, and **A10**, in which N coordinates at the O5 position, expectedly have a highly asymmetric NQI tensor (*η* > 0.8), due to the bonding with Mn4, Mn3, and Mn1 (Figures 3). In all other models ammonia binds as a terminal ligand, thus the NQI asymmetry depends mostly on the hydrogen bonding environment around NH_3_. Ammonia in the W1 position has 3 unequal H-bonding interactions with Ser169, Asp61, and a distant water molecule (Figures 4–6), whereas in the W2 position, it interacts with only two water molecules. The calculated asymmetry is high (*η* > 0.8) for **B1**, **B3,** and **D4**, whereas a lower degree of asymmetry is predicted for **C1**, **C2**, **D5,** and **E1**–**E3**, for which η is between 0.2 and 0.7. Overall, only models **C1**, **C2,** and **D5** reproduce both the unchanged ^14^N His332 HFC and the ^14^N NH_3_ NQI tensor anisotropy.

#### 3.4.3 ^17^O HFCs

The calculated and experimental ^17^O HFCs are given in Tables 4 and S5. Experimentally, three types of ^17^O HFCs are observed in the untreated S_2_ state, one with large |*A*_iso_| attributed to O5, and two with smaller |*A*_iso_| values attributed to terminal water or hydroxo ligands.^45^ Upon treatment with ammonia, the line intensity of the smallest |*A*_iso_| decreases significantly, which has been linked to the loss of a water terminal ligand. Models **A5**, **A8**, and **A9** have only small (<4 MHz) ^17^O HFCs, due to the absence of the exchangeable bridging O ligand. By contrast, **A4**, **D4**, and **D5** have ^17^O HFCs with |*A*_iso_| significantly larger (>16 MHz) compared to the experimental value of 7 MHz. Overall, models **B1**, **B3**, **C2,** and **E1**–**E3** are most consistent with experimental ^17^O HFCs. Among those, **B1** and **B3** also exhibit smaller ^17^O isotropic HFC for O5 compared to the S_2_ state models, even though they do not reproduce the reported^43^ ∼30% reduction.

**Table 4:** Calculated Effective/Projected ^17^O Isotropic Hyperfine Coupling Constants of the W1, W2, and O5 Ligands of the S_2_–NH_3_ Models with *S*_GS_ = 1/2^a^

	O5	W1	W2
**A4**		3.8	16.7
**A5**		0.7	0.6
**A8**		1.2	3.9
**A9**		0.9	0.4
**A10**		8.2	0.5
**B1**	**8.1**		**4.7**
**B3**	**10.8**		**1.4**
**C1**	11.9	8.8	
**C2**	**12.2**	**3.4**	
**D4**	8.2	16.8	12.3
**D5**	33.9	3.6	0.9
**E1**	**4.8**	**5.5**	**5.4**
**E2**	**8.6**	**2.4**	**11.1**
**E3**	**11.5**	**2.6**	**1.7**
*Exp.*			
*T. vestitus*(45)	7.0	3.1	
S_2_ state without ammonia:			
**S**_**2**_	8.5	9.4	1.3
**S**_**2**_^**H**^	12.0	1.2	2.5
*Exp.*			
*T. vestitus*(45)	9.7	4.5	1.4

a Results are compared with parameters fitted from W-band EDNMR spectra.

To summarize, among the 14 *S*_GS_ = 1/2 models studied, **B3** and **C2** are the most consistent with experimentally derived EPR parameters.

### 3.5 Redox Properties

**Figure 9:**
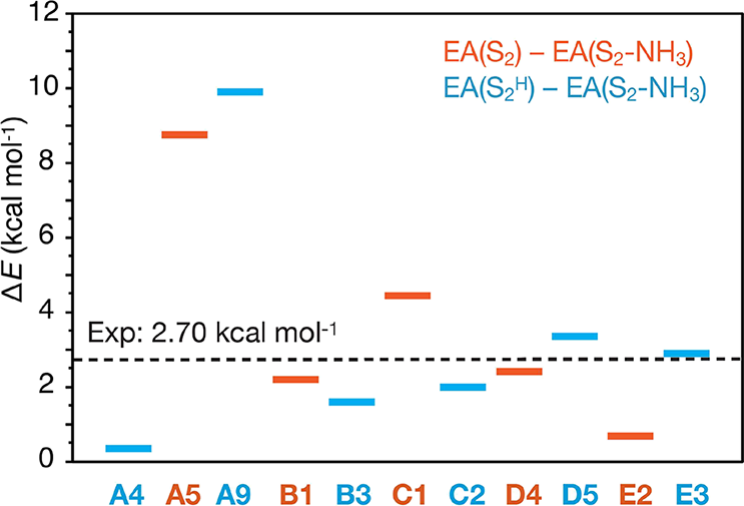
Electron affinity (EA) differences between S_2_–NH_3_ and the S_2_-state models, i.e., Δ*E* = EA(S_2_) – EA(S_2_–NH_3_). Comparisons are made between structures having the same charge; thus, models in blue are compared with S_2_^H^ and models in orange are compared with S_2_. The plotted values are listed in Table S6.

In addition to agreement with spectroscopic observations, the electronic structure of an S_2_–NH_3_ model must be able to follow the experimentally observed S-state progression. Vinyard et al.^81^ showed that the one-electron reduction of the ammonia-bound S_2_ state to S_1_ is ∼50% slower than that of the untreated S_2_ state. They estimated the value of the difference between the electron affinities of S_2_ and S_2_–NH_3_ to be around 2.70 kcal mol^–1^ or higher. In order to examine which of our models are consistent with this observation, we computed their electron affinities. The differences between the electron affinities of the ammonia-bound S_2_ and the untreated S_2_ state are plotted in Figure 9. We note that the differences refer to structures with the same total charge, thus for models **A4**, **B3**, **C2**, **D5,** and **E3** the EA differences from the S_2_ state with W2 protonated in the aquo form are reported, whereas for models **A5**, **B1**, **C1**, **D4,** and **E2** the differences from the S_2_ state with W2 in the hydroxo form are reported. Models **A8**, **A10**, and **E1** are not shown, because they have a different charge (different total number of protons) than S_2_ and S_2_^H^.

Figure 9 shows that all S_2_–NH_3_ models have smaller EAs than S_2_ state models, as in the experiment. The EAs of **B1**, **B3**, **C2**, **D4**, **D5,** and **E3** relative to the S_2_ state are very close (within 1.3 kcal mol^–1^) to the experimentally determined value of 2.70 kcal mol^–1^. The EAs of **A5** and **A9** are much larger, 8.7 and 9.9 kcal mol^–1^, respectively, whereas of **A4** and **E2** they are less than 1 kcal mol^–1^. It is important to note that all calculated EA differences are so small that fall within the limits of the accuracy of DFT, thus all models can be considered consistent with this criterion. Nevertheless, these results serve to remove the main argument against substitution modes B and C, namely that their EAs would be almost the same as that of the untreated S_2_ state.^81^

For completeness, we finally examine whether the binding of ammonia disrupts the redox balance of the OEC to the extent that (physiological) formation of the immediate next step, i.e., oxidation of the Yz radical,^120−122^ is inhibited. Therefore, we examined the electronic structure of one-electron oxidized S_2_–NH_3_ models. The locus of oxidation of all models is indeed Yz, as indicated by the calculated spin density distribution upon oxidation. Notably, an inspection of the canonical molecular orbitals indicates that the HOMO is localized on Yz in all models, except **A8**, **C1,** and **E1**, whose HOMO is located mostly on O5, Asp61, and W1/W2 ligands, respectively. However, the spin density upon oxidation is on Yz^•^ as well, indicating that oxidation of all S_2_–NH_3_ models would be physiologically mediated by the redox-active Yz residue (Figure S2). These results suggest that for all examined models the oxidation of Yz is not inhibited, therefore ammonia (in the noninhibitory binding mode) does not have to detach in order for the OEC to form the S_2_Yz^•^ state. Whether ammonia remains bound to the OEC also in the S_3_ state, as implied by previous studies,^69,123,124^ cannot be confirmed with the present models and remains an open question.

## 4 Discussion

### 4.1 Ammonia Coordination in the S_2_ State

In the previous section, we presented a systematic screening of ammonia-bound S_2_ state OEC models against available experimental data. We optimized 29 S_2_–NH_3_ models, representative of previously suggested ammonia binding modes, and calculated their relative energies, magnetic/spectroscopic properties, and redox behavior. Given that it is not possible to define based on which a model is excluded, the results serve to distinguish the models that demonstrate closer alignment with experimental observations in comparison to others.

Substitution of the O5 μ-oxo bridging ligand by ammonia is clearly inconsistent with the majority of experimental data. Models with binding mode A can be ruled out on energetic grounds, as well as due to the highly anisotropic ^14^N hyperfine tensors and absence of an O ligand with predicted ∼7 MHz *A*_iso_. A model similar to **A4** has been previously investigated computationally by Schraut and Kaupp.^68^ Both **A4** and the previously reported^68^ model have very similar structural parameters, differing only in the protonation state of the W1 ligand. Both studies confirm the disagreement of the calculated nuclear quadrupole asymmetry parameter as well as that the amido bridging ligand is strongly energetically unfavorable compared to W1 substitution. Furthermore, in a computational study, Guo et al.^125^ showed that direct W1 replacement by ammonia is also kinetically favored, with a transition state in the order of 10 kcal mol^–1^, in stark contrast to O5 substitution that was shown to be thermodynamically forbidden with a transition state higher than 30 kcal mol^–1^. An argument in favor of binding motif A has been the loss of a vibrational mode at 606 cm^–1^ upon ammonia binding,^66^ but it has already been shown, using a model that corresponds to model **B1** of the present work, that this feature can be reproduced by ammonia binding on the W1 position.^43^ Moreover, the calculated electron affinities of models **A4**, **A5** and **A9** are far from those estimated from experiment, with **A4** being almost the same as the untreated S_2_, and **A5** and **A9** being ∼6 kcal mol^–1^ lower than the experimentally estimated value. Therefore, the results reported herein using our refined larger models are in agreement with previous works,^43,45,68^ favoring terminal water substitution against O5 substitution.

Models **D4** and **D5**, which represent ammonia binding as an additional ligand on Mn4 (binding mode D), are inconsistent with both ^55^Mn and ^17^O HFCs. Besides, we located structural isomers, **D2** and **D7** respectively, which are lower in energy and predicted to have high-spin ground states (Figure 7), suggesting that ammonia-treated S_2_ state samples would have to exhibit high-spin EPR signal(s) if binding mode D was taking place. Similarly, S_2_–NH_3_ models that represent ammonia coordination to Mn1, **E1**, **E2,** and **E3**, are inconsistent with ^55^Mn and ^14^N HFCs and are higher in energy than their high-spin isomers **D2** and **D7**, respectively.

Models **B1**, **B3**, **C1**, and **C2**, which represent terminal W ligand substitution by ammonia, are in best agreement with the majority of experimental data. The model that simultaneously satisfies all the evaluation criteria is W2 substitution by NH_3_, model **C2**. Model **B3** (W1 substitution by NH_3_) is also consistent with all experimental observations, except for the overestimation of the nitrogen nuclear quadrupole coupling asymmetry. Importantly, W1 substitution is also supported by mutation studies. Oyala et al.^46^ reported that mutation of the amino-acid D1-Asp61 (Figure 5) to the non-hydrogen bonding residue alanine, leaves the ammonia ^14^N hyperfine couplings unaltered with respect to the native D1-Asp61 PSII, but at the same time the NQI asymmetry is dramatically reduced, from 0.42 to 0.04. These results suggest that the nuclear quadrupole coupling asymmetry arises from and is thus very sensitive to the hydrogen-bonding network around the coordination site. Besides, the wide range (0.4–0.6) of experimentally determined *η* values that have been observed for different organisms^45,46,58^ might be attributed to differences in amino-acid chains located far from the active site, which has been recognized^47,126^ to perturb the geometry of the OEC, particularly around Mn4. We note that it is hard to fully account for long-range hydrogen-bonding effects in quantum chemical models. Indeed, the calculated asymmetries for models similar to our **B1** span a relatively wide range of values (0.35–0.87), despite models being similar in other computed parameters (Tables S7 and S8), which underlines the high sensitivity of this parameter to the structure of the hydrogen-bonding network. Therefore, both **B3** and **C2** can be considered as the most consistent with the entirety of available experimental data.

Considering the preferred protonation state of the terminal W1/W2 ligand, models **B1** and **C1** that have a hydroxo ligand on Mn4 are less consistent with the experimental ^55^Mn HFCs than **B3** and **C2**, respectively, in which the Mn4 ligand is in the aquo form. Thus, our results suggest that the remaining water ligand on Mn4 is protonated in the aquo form. We stress that there is no experimental constraint regarding specifically the protonation states of terminal water ligands, as ammonia binding and inhibition are independent of the solvent pH.^69,127^ This can be explained if the concentration of ammonia near the OEC remains unaffected by that within the bulk solution. This scenario might occur if ammonium undergoes deprotonation *after* entering the PSII, by a residue that would otherwise remain deprotonated.^69^ Overall, our results exclude binding modes A, D, and E, and favor terminal W1/W2 ligand substitution that corresponds to modes B and C.

### 4.2 Implications for the O–O Bond Formation Mechanism

**Figure 10:**
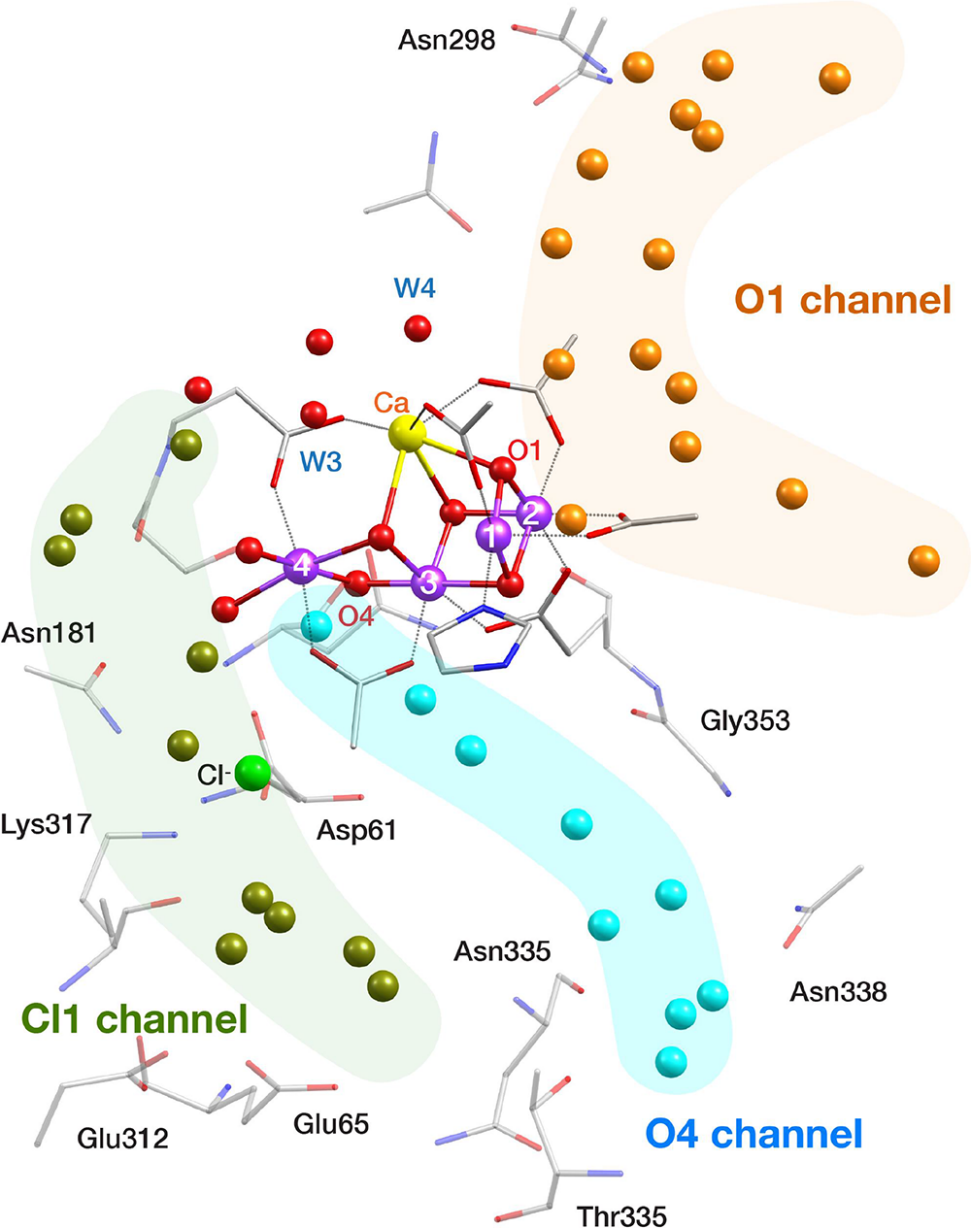
Crystallographic structure of the S_1_ state of the OEC (PDB ID 5B66, monomer A)^75^ with the water channels shown in different colors.

The most important implication of water analogue binding on the OEC is how it relates to the binding pathway and identity of the water substrates for the subsequent O–O bond formation. Water transport and proton release during the water oxidation reaction are mediated by water channels that begin near the OEC and extend toward the environment of PSII. Three water channels have been identified and are shown in Figure 10.^31−39^ The Cl1 channel, which originates near Mn4, has been associated with proton release during the S_2_ → S_3_ and S_3_ → [S_4_] transitions.^22,37,128−135^ Several mechanistic suggestions have been based on the hypothesis of substrate delivery either from the O4^122,136^ or from the O1 channels,^137−144^ which begin near the O4 and O1 bridging-oxo ligands, respectively. Assuming that ammonia insertion leading to the EPR consistent models identified in this work proceeds analogously with water insertion, two different binding pathways can be considered that involve the O4 and the O1 channels, respectively.

In the first pathway, ammonia may be considered to approach Mn4 from the O4 channel and bind to the W1 or W2 positions (models **B3** and **C2**). In the second pathway, ammonia accesses the OEC from the O1 channel, interacts with Ca^2+^, but eventually moves to the Mn4 as the most thermodynamically stable binding site, leading to exactly the same models. It is conceivable that either one or both of these pathways can operate simultaneously.

The investigation of second sphere interactions does not allow us to determine the optimal ammonia approach pathway. Mandal et al.^55^ carried out a computational sampling of ammonia binding sites in the second coordination sphere of the OEC to determine the “secondary” ammonia binding site and found six high-affinity sites all of which are energetically close. Two binding sites were found near Mn4 and a third in which ammonia is hydrogen bonded to W3. Complementary insight may be obtained from spectroscopic studies using ^13^C labeled methanol, another water analogue that has been used to probe substrate binding to the OEC. Oyala et al.^44^ identified three possible interaction areas of ^13^C labeled methanol with the Mn_4_CaO_5_ cluster, which include the W3 site and two second coordination sphere sites in the proximity of O4 and O1. In a subsequent study by Nagashima and Mino,^49^ the possible locations of methanol were associated with the proximal region of the O1 channel close to Mn1 as well as with a region near Mn4. In a computational study, Retegan and Pantazis^47^ reported that noncovalent binding of methanol at the end point of the O4 channel is most consistent with the spectroscopic observations of Oyala et al.^44^ Moreover, they proposed^47,50^ that the higher accessibility of methanol to the OEC of higher-plant versus cyanobacterial PSII is due to the difference in the O4 channel width caused by a single amino acid replacement at the end of the O4 channel, specifically D1-Asn87 in cyanobacteria is replaced by alanine in higher plants.^145^ Interestingly, mutation of the Asn87 residue to alanine in *Synechocystis sp.* PCC 6803 enables methanol coordination to Mn4 at the W2 site, as shown by ^13^C hyperfine spectroscopy experiments combined with QM/MM calculations.^146^ These studies favor the O4 insertion pathway for methanol, without necessarily excluding the O1 channel as an alternative approach pathway. We suppose that ammonia approaching from the O1 channel would require more extensive reorganization of the hydrogen bonding network around the OEC in order to eventually bind to Mn4, but a proper computational investigation of this question would require costly ab initio molecular dynamics calculations that are currently inaccessible.

A final question is whether we can correlate the ammonia-bound models discussed above with the inhibition of the OEC catalytic activity. This is particularly important in light of the recent extensive studies by Dau and co-workers^69,74^ which showed that there are at least two different S_2_–NH_3_ species with different O_2_ evolution activities. Specifically, time-resolved O_2_ polarography, recombination fluorescence and FTIR difference spectra on PSII showed that the slower O_2_ evolution of ammonia-treated samples is attributed to complete inhibition of O_2_ formation in only a fraction (∼50%) of samples, rather than to slowed O_2_ formation. Schuth et al.^69^ proposed an equilibrium between W1 and W2 substituted structures, with the former being active and the latter inactive.

One possibility for models **B3** and **C2** favored by the present study is that they both represent noninhibitory ammonia binding. Otherwise, one of them could represent an inhibited S_2_–NH_3_ state. The small energy difference between models **B3** and **C2** (1.4 kcal mol^–1^) implies that the two species can coexist. This is in line with the results of Schuth et al.^69^ that indicate similar ammonia binding constants for both inhibitory and noninhibitory sites. Interestingly, the calculated ^55^Mn, ^14^N, and ^17^O HFCs of **B3** and **C2** are very similar (Tables 2–4), implying that they would be hardly discernible by magnetic resonance spectroscopies. Therefore, under the assumption that one of **B3** or **C2** is inhibitory, our calculations are consistent with the equilibrium between W1 and W2 substituted structures suggested by Schuth et al.^69^

Two mechanisms of oxygen evolution inhibition by terminal W1/W2 ligand substitution by ammonia can be considered, one that involves replacing a substrate oxygen ligand and another in which ammonia blocks proton transfer from the OEC to the Cl1 channel, inhibiting a deprotonation event required for S-state progression. While the assignment of the oxygen evolution inhibitory and noninhibitory binding sites to W1 and W2 cannot be made conclusively with the available experimental and computational data, we can consider the following scenarios. First, if W2 is a substrate,^26−28,113,122,147,148^ then ammonia binding at the W2 site (model **C2**) is inhibitory, whereas binding at the W1 site (model **B3**) is noninhibitory. This implies that proton transfer during the deprotonation events of the S_2_→ S_3_ and possibly S_3_ → S_4_ transitions is not mediated by W1. Exactly the reverse argument holds in the unlikely case that W1 is a substrate, where the noninhibitory assignment of ammonia binding to the W2 site would imply that W1 mediates proton transfer. In both cases, the substrate would also be the group that mediates deprotonation that enables S-state progression. If, on the other hand, neither of W1 or W2 is a substrate, then in case either one of the models **B3** or **C2** represents an inhibited state of the OEC, the inhibition mechanism arises either from disruption of the requisite deprotonation step irrespective of the group (W1 or W2) that mediates it physiologically or from blocking the insertion of the substrate (in this case, W3)^27,135,139,141,142,144,149^ in the S_2_ → S_3_ transition.

## 5 Conclusions

The results presented herein serve to distinguish models for ammonia binding to the S_2_ state of the OEC that align with experimental observations. Ammonia-bound models in the S_2_ state were optimized and compared with respect to their magnetic and spectroscopic properties, relative energies, and redox potentials. We examined several variants of ammonia-bound models with different ammonia binding modes, including O5 substitution, terminal ligands W1 or W2 substitution, Mn4 addition as a sixth ligand in the closed-cubane conformation of the Mn_4_CaO_5_ cluster and Mn1 addition as a sixth ligand in the open-cubane conformation. Our results extend and elaborate on past computational results regarding experimentally consistent types of substitution models, while at the same time providing important new data on the spectroscopic validity of newly proposed ammonia addition possibilities. Substitution of the bridging μ-oxo ligand O5 by ammonia is found less favorable based on energetic criteria and ligand hyperfine coupling constants. Similarly, the addition of ammonia as a sixth ligand on Mn4 in the closed cubane conformation or on Mn1 in the open cubane conformation of the Mn_4_CaO_5_ cluster results in larger deviations from the experimental hyperfine coupling parameters. By contrast, two binding modes that involve ammonia coordination as a terminal ligand on Mn4 replacing either W1 or W2 are found to be most consistent with experimental observations. These results are in line with recent experiments showing an equilibrium between a functional and nonfunctional ammonia-bound species and therefore point toward an equilibrium between species in which ammonia binds on the W1 and W2 positions.

## Supporting Information Available

QM model of the S_2_ state; structural parameters of the geometry optimized models; Mulliken Mn spin populations; relative energies; effective ^55^Mn HFCs, on-site hyperfine values and spin projection coefficients; effective ^14^N HFCs, NQI tensors, and ^17^O hyperfine tensors; electron affinity (EA) differences between S_2_–NH_3_ and the S_2_-state models; frontier orbitals of **C1** and **C1Y**_*z*_^**•**^; comparison of calculated ^55^Mn HFCs with those of previous studies; comparison of calculated ^14^N HFCs and NQI asymmetry with those of previous studies; and Orca input files (PDF)
Cartesian coordinates of all structural models discussed in this work (TXT)
Orca input files (TXT)
