# Supplementary material for: Comprehensive Evaluation of Models for Ammonia Binding to the Oxygen Evolving Complex of Photosystem II
Source: J Phys Chem B. 2024 Feb 1;128(6):1333–49. doi: 10.1021/acs.jpcb.3c06304 (PMC10875651; doi:10.1021/acs.jpcb.3c06304)
Supplement: Supplementary file 1 — jp3c06304_si_001.pdf [file jp3c06304_si_001.pdf]

Supporting information

for

Comprehensive Evaluation of Models for Ammonia Binding to the Oxygen Evolving Complex of Photosystem II

Maria Drosou,[a,b,*] Dimitrios A. Pantazis[a,*]

[a] Max-Planck-Institut für Kohlenforschung Kaiser-Wilhelm-Platz 1, 45470, Mülheim an der Ruhr, Germany

[b] Inorganic Chemistry Laboratory, National and Kapodistrian University of Athens, Panepistimiopolis, Zografou 15771, Greece

E-mail: drosou@kofo.mpg.de, dimitrios.pantazis@kofo.mpg.de

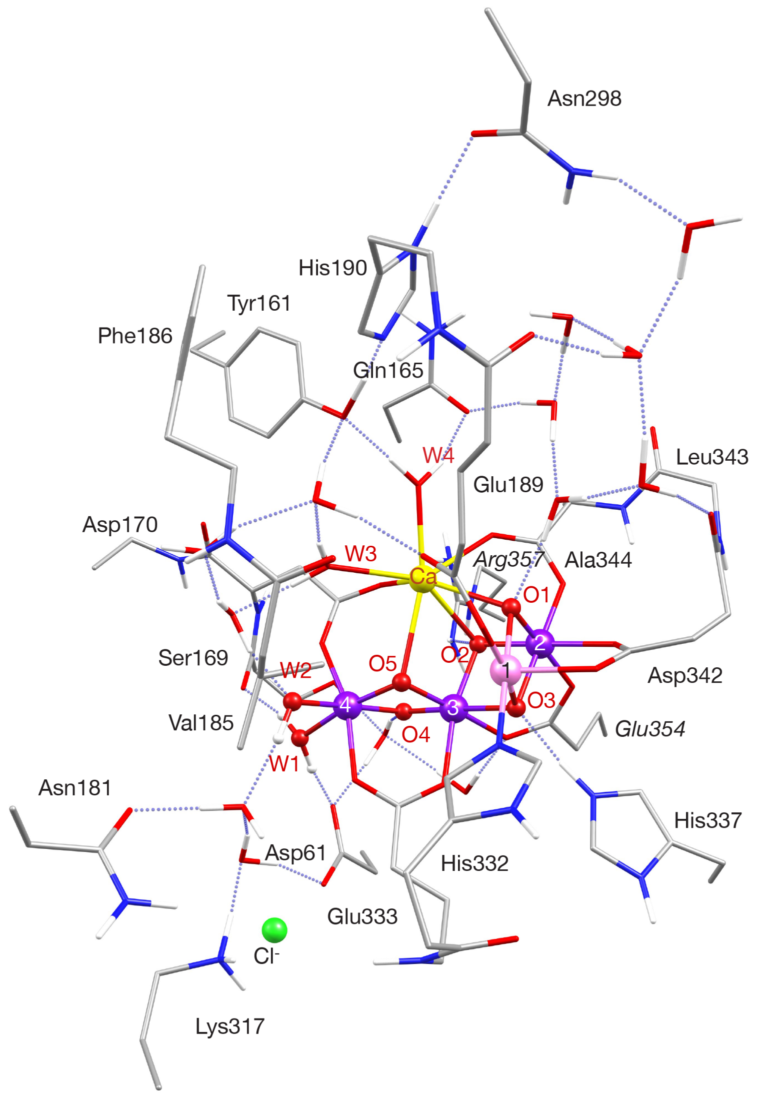

Figure S1. Complete computational model for the S_2 state of the OEC. Mn(III) ions are indicated in pink, Mn(IV) in dark purple, Ca in yellow, N in blue, O in red and Cl in green. Hydrogen atoms connected to carbon atoms are omitted for clarity. All amino-acid residues belong to the D1 subunit, except from Arg357 and Glu354 (shown in *italics*), which belong to the CP43 subunit.

Table S1. Structural parameters of BP86/ZORA-TZVP optimized S_2-state structures with various NH_3 binding sites. The corresponding core structures are shown in Figures 4–6 of the manuscript.

	Mn1-Mn2	Mn1-Mn3	Mn2-Mn3	Mn3-Mn4	Mn[a]-NHx	Mn1-N(His)	Mn3-O5	Mn[b]-O5	Mn4-O(W1)	Mn4-O(W2)
**S_2**	2.765	3.386	2.814	2.768	-	2.022	1.846	1.881	2.032	1.862
**S_2^H**	2.772	3.373	2.800	2.755	-	2.016	1.860	1.861	2.008	2.039
**A1**	2.751	3.618	2.901	3.023	1.932	2.041	-	1.803	1.957	2.231
**A2**	2.797	3.565	2.833	2.866	1.885	2.041	-	2.139	2.002	2.066
**A3**	2.765	3.362	2.850	2.758	1.881	2.021	-	-	2.046	2.068
**A4**	2.757	3.412	2.830	2.903	2.005	2.021	-	-	1.968	1.850
**A5**	2.797	3.426	2.825	2.714	1.815	2.056	-	-	2.112	2.078
**A6**	2.764	3.382	2.813	2.804	1.924	2.031	-	-	2.071	1.867
**A7**	2.770	3.374	2.798	2.793	1.914	2.024	-	-	2.044	2.039
**A8**	2.770	3.271	2.835	2.695	1.879	2.033	-	-	2.198	1.890
**A9**	2.771	3.300	2.812	2.683	1.843	2.029	-	-	2.161	2.069
**A10**	2.768	3.387	2.809	2.892	2.009	2.009	-	-	1.946	2.015
**B1**	2.766	3.350	2.810	2.771	2.061	2.025	1.832	1.917	-	1.869
**B2**	2.742	2.923	2.782	3.112	2.182	2.055	1.888	1.884	-	1.866
**B3**	2.773	3.342	2.799	2.759	2.059	2.018	1.848	1.891	-	2.039
**B4**	2.745	2.907	2.770	3.150	2.167	2.050	1.880	1.883	-	2.035
**C1**	2.766	3.360	2.807	2.773	2.056	2.025	1.812	1.985	1.814	-
**C2**	2.769	3.391	2.801	2.748	2.060	2.017	1.862	1.859	1.997	-
**C3**	2.745	2.909	2.768	3.118	2.062	2.052	1.884	1.886	2.114	-
**D1**	2.748	3.118	2.835	3.205	2.077	2.057	2.254	1.774	2.155	1.786
**D2**	2.787	3.595	2.854	2.882	2.050	2.062	1.858	1.857	-	2.280
**D3**	2.747	3.147	2.843	3.276	2.004	2.057	2.263	1.785	1.895	1.958
**D4**	2.786	3.533	2.826	2.830	2.043	2.064	1.852	1.879	-	1.871
**D5**	2.777	3.154	2.783	3.260	1.985	2.025	1.898	2.208	2.091	1.888
**D6**	2.749	3.193	2.831	3.232	2.030	2.051	2.210	1.873	2.112	1.861
**D7**	2.757	3.755	2.917	2.959	2.014	2.043	1.958	1.915	-	2.072
**D8**	2.747	3.096	2.819	3.287	1.981	2.056	2.230	1.779	1.940	2.125
**E1**	2.798	3.427	2.829	2.805	2.415	2.082	1.789	1.953	1.834	1.857
**E2**	2.857	3.548	2.842	2.803	2.018	2.359	1.859	1.848	2.001	1.872
**E3**	2.804	3.410	2.813	2.762	2.430	2.065	1.847	1.827	1.995	2.062
**E4**	2.783	3.572	2.853	2.789	1.980	2.066	1.843	1.875	1.975	2.072

[a]Mn4 ion in binding modes A–D and Mn1 in mode E.

[b]Mn1 ion for closed-cubane structures and Mn4 ion for open-cubane structures.

Table S2. Calculated Mulliken Mn spin populations for the optimized S_2 and S_2-NH_3 models calculated with broken-symmetry (BS) TPSSh using the lowest energy BS determinant. Very similar spin populations are computed with BP86 and with TPSSh using the high-spin determinant.

	Mn1	Mn2	Mn3	Mn4
**S_2**	3.90	-2.96	-2.90	2.91
**S_2^H**	3.89	-2.96	-2.93	2.92
**A1**	2.96	2.99	2.79	-3.99
**A2**	-3.89	2.94	3.12	3.10
**A3**	-3.88	2.97	2.99	3.02
**A4**	3.88	-2.98	-2.89	2.97
**A5**	3.92	-2.94	-2.84	2.73
**A6**	-3.90	2.95	2.99	3.02
**A7**	-3.88	2.95	3.03	3.03
**A8**	3.91	-2.92	-2.75	2.79
**A9**	3.90	-2.94	-2.80	2.70
**A10**	3.86	-2.97	-2.93	2.98
**B1**	3.94	-2.97	-2.92	2.96
**B2**	3.03	2.93	2.96	3.84
**B3**	3.89	-2.96	-2.93	2.95
**B4**	2.99	2.93	2.99	3.90
**C1**	3.90	-2.94	-2.89	2.84
**C2**	3.89	-2.96	-2.92	2.94
**C3**	2.97	2.93	2.87	-3.90
**D1**	2.87	2.91	-3.84	2.79
**D2**	2.91	2.96	2.78	-3.95
**D3**	2.88	2.90	-3.83	2.92
**D4**	3.90	-2.93	-2.93	2.95
**D5**	3.88	-2.90	2.95	-2.89
**D6**	2.98	2.90	-3.84	2.86
**D7**	2.99	2.99	3.89	-2.81
**D8**	2.88	2.91	-3.82	2.77
**E1**	3.92	-2.93	-2.83	2.87
**E2**	3.90	-2.88	-2.93	2.91
**E3**	3.92	-2.95	-2.93	2.90
**E4**	2.97	-2.96	-2.81	3.91

Table S3. Relative energies (kcal mol^{-1}) of BP86 optimized S_2-state structures with various NH_3 binding sites calculated with BP86, TPSSh using the high-spin determinant and broken-symmetry (BS) TPSSh using the lowest energy BS determinant.

(O,H)		BP86	TPSSh	BS-TPSSh
(2,4)	**A6**	15.0	19.6	19.4
	**A9**	24.4	26.7	25.5
	**B1**	0.0	0.0	0.0
	**B2**	8.3	10.4	11.1
	**C1**	1.3	6.5	6.4
(2,5)	**A4**	32.5	39.9	39.9
	**A7**	20.0	21.9	21.7
	**B3**	3.5	1.3	1.4
	**B4**	8.6	5.2	6.0
	**C2**	0.0	0.0	0.0
	**C3**	5.1	4.6	5.1
(3,6)	**A5**	23.0	27.3	26.2
	**D1**	9.8	12.5	12.2
	**D2**	0.0	0.0	0.0
	**D3**	10.3	13.8	12.7
	**D4**	7.0	5.9	5.1
	**E2**	15.1	20.4	19.6
(3,7)	**A1**	24.1	26.8	26.4
	**A2**	33.2	36.9	36.4
	**A3**	34.6	45.0	45.5
	**D5**	10.9	13.4	13.7
	**D6**	3.9	8.0	8.2
	**D7**	0.0	0.0	0.0
	**D8**	9.1	14.1	12.8
	**E3**	5.0	7.5	6.7
	**E4**	9.9	16.1	13.8

Table S4. Calculated effective/projected axial ($A_{||}$) and equatorial ($A_{\perp}$) ^{55}Mn hyperfine coupling constants, as well as isotropic ($a_{||}$) and anisotropic ($a_{\perp}$) on-site hyperfine values and spin projection coefficients ρ_i for each Mn ion, Mn$_i$, of the S_2-NH_3 and the S_2-state models in the low-spin (S = 1/2) configuration.

		Mn1(III)	Mn2(IV)	Mn3(IV)	Mn4(IV)
**A4**	$A_{BS,x}$ [a]	-179	402	369	-438
	$A_{BS,y}$	-468	415	377	-469
	$A_{BS,z}$	-534	440	437	-498
	$A_{\|\|}$ [b]	-85	-199	-240	-419
	$A_{\perp}$ [c]	-239	-185	-205	-463
	ρ_i	1.07	-0.76	-0.92	1.61
	α_{iso} [d]	-175	249	234	-278
	α_{aniso} [e]	-143	-19	-38	-27
**A5**	$A_{BS,x}$	-265	397	404	-450
	$A_{BS,y}$	-579	407	460	-524
	$A_{BS,z}$	-605	447	471	-542
	$A_{\|\|}$	-229	-265	-173	-210
	$A_{\perp}$	-510	-238	-199	-248
	ρ_i	1.94	-1.00	-0.72	0.78
	α_{iso}	-215	247	264	-300
	α_{aniso}	-145	-26	37	-49
**A8**	$A_{BS,x}$	-154	418	398	-427
	$A_{BS,y}$	-454	425	430	-525
	$A_{BS,z}$	-491	461	444	-539
	$A_{\|\|}$	-133	-273	-201	-209
	$A_{\perp}$	-407	-250	-187	-260
	ρ_i	1.94	-1.00	-0.76	0.82
	α_{iso}	-163	258	252	-295
	α_{aniso}	-142	-23	-18	-62
**A9**	$A_{BS,x}$	-172	407	404	-446
	$A_{BS,y}$	-464	417	463	-525
	$A_{BS,z}$	-522	452	473	-549
	$A_{\|\|}$	-151	-266	-156	-177
	$A_{\perp}$	-433	-242	-181	-213
	ρ_i	1.97	-0.99	-0.65	0.67
	α_{iso}	-172	253	265	-301
	α_{aniso}	-143	-24	38	-54
**A10**	$A_{BS,x}$	-211	393	389	-429

	$A_{BS,y}$	-476	404	400	-470
	$A_{BS,z}$	-579	431	446	-495
	$A_{\|\|}$	-96	-210	-220	-416
	$A_{\perp}$	-238	-194	-194	-468
	ρ_i	1.01	-0.82	-0.83	1.63
	a_{iso}	-188	243	244	-276
	a_{aniso}	-141	-19	-31	-32
**B1**	$A_{BS,x}$	-167	404	369	-426
	$A_{BS,y}$	-463	416	384	-466
	$A_{BS,z}$	-521	447	421	-503
	$A_{\|\|}$	-135	-263	-221	-315
	$A_{\perp}$	-398	-241	-198	-279
	ρ_i	1.82	-0.99	-0.89	1.06
	a_{iso}	-171	251	232	-276
	a_{aniso}	-144	-22	-27	-35
**B3**	$A_{BS,x}$	-176	392	390	-424
	$A_{BS,y}$	-466	405	417	-471
	$A_{BS,z}$	-535	437	449	-492
	$A_{\|\|}$	-131	-251	-261	-320
	$A_{\perp}$	-373	-229	-234	-364
	ρ_i	1.68	-0.97	-0.98	-0.98
	a_{iso}	-174	244	248	-274
	a_{aniso}	-144	-23	-27	-34
**C1**	$A_{BS,x}$	-167	406	369	-450
	$A_{BS,y}$	-461	417	378	-461
	$A_{BS,z}$	-521	450	407	-498
	$A_{\|\|}$	-147	-262	-147	-179
	$A_{\perp}$	-434	-240	-135	-164
	ρ_i	1.99	-0.98	-0.61	0.61
	a_{iso}	-170	252	228	-279
	a_{aniso}	-144	-23	-20	25
**C2**	$A_{BS,x}$	-175	397	390	-442
	$A_{BS,y}$	-463	410	416	-476
	$A_{BS,z}$	-533	441	449	-483
	$A_{\|\|}$	-123	-247	-264	-354
	$A_{\perp}$	-352	-226	-238	-384
	ρ_i	1.59	-0.94	-0.99	1.35
	a_{iso}	-174	247	248	-277

	α_{aniso}	-144	-23	-27	-22
**D4**	$A_{BS,x}$	-368	386	349	-419
	$A_{BS,y}$	-679	399	375	-453
	$A_{BS,z}$	-730	437	426	-500
	$A_{\parallel}$	-204	-196	-252	-447
	$A_{\perp}$	-391	-177	-214	-390
	ρ_i	1.25	-0.76	-1.00	1.51
	α_{iso}	-264	242	228	-271
	α_{aniso}	-150	-26	-38	38
**D5**	$A_{BS,x}$	-311	471	-430	397
	$A_{BS,y}$	-589	481	-447	422
	$A_{BS,z}$	-675	503	-509	464
	$A_{\parallel}$	-203	-231	-376	-258
	$A_{\perp}$	-412	-219	-324	-228
	ρ_i	1.47	-0.77	1.25	-0.94
	α_{iso}	-234	288	-274	254
	α_{aniso}	-143	-16	42	-33
**E1**	$A_{BS,x}$	-291	408	355	-475
	$A_{BS,y}$	-603	417	362	-484
	$A_{BS,z}$	-624	452	392	-503
	$A_{\parallel}$	-257	-267	-106	-139
	$A_{\perp}$	-542	-244	-97	-132
	ρ_i	1.99	-1.00	-0.45	0.46
	α_{iso}	-225	252	219	-289
	α_{aniso}	-143	-24	-20	14
**E2**	$A_{BS,x}$	-315	371	362	-437
	$A_{BS,y}$	-556	394	389	-473
	$A_{BS,z}$	-679	428	436	-512
	$A_{\parallel}$	-207	-218	-256	-417
	$A_{\perp}$	-405	-195	-220	-370
	ρ_i	1.47	-0.86	-0.99	1.37
	α_{iso}	-230	236	235	-281
	α_{aniso}	-135	-27	-36	34
**E3**	$A_{BS,x}$	-287	388	386	-439
	$A_{BS,y}$	-601	399	419	-481
	$A_{BS,z}$	-622	432	452	-493
	$A_{\parallel}$	-197	-228	-224	-343

	$A_\perp$	-421	-208	-252	-381
	ρ_i	1.55	-0.89	-0.98	1.32
	a_{iso}	-224	241	249	-280
	a_{aniso}	-145	-23	29	-28
**S_2**	$A_{BS,x}$	-165	405	366	-437
	$A_{BS,y}$	-459	418	385	-479
	$A_{BS,z}$	-519	449	423	-515
	$A_\parallel$	-131	-261	-227	-287
	$A_\perp$	-387	-239	-202	326
	ρ_i	1.78	-0.98	-0.91	1.11
	a_{iso}	-170	251	232	-283
	a_{aniso}	-144	-22	-28	-35
**S_2^H**	$A_{BS,x}$	-175	393	386	-443
	$A_{BS,y}$	-463	407	415	-485
	$A_{BS,z}$	-534	438	447	-494
	$A_\parallel$	-127	-249	-262	-344
	$A_\perp$	-363	-227	-235	-380
	ρ_i	1.64	-0.96	-0.99	1.31
	a_{iso}	-174	245	247	-281
	a_{aniso}	-144	-22	-28	-27

[a] $A_{BS,x}$, $A_{BS,y}$, $A_{BS,z}$ are the components of the hyperfine coupling tensor computed from the BS calculation and following the convention $|A_{BS,z}| > |A_{BS,y}| > |A_{BS,x}|$. The effective (observable) HFC components –A_x, A_y, A_z– of Mn$_i$ are derived from: $A_x = A_{BS,x} \cdot \frac{\langle S_z \rangle_{BS}}{S_i} \cdot \rho_i \cdot 1.78$, where S_i is the site spin of Mn$_i$ [2 for Mn(III) and 3/2 for Mn(IV)], $\langle S_z \rangle_{BS}$ the total M_S of the BS wavefunction (1/2 in all models), ρ_i the spin projection coefficient of Mn$_i$,[1] and 1.78 is a scaling factor used specifically for comparing the computed ^{55}Mn hyperfine coupling constants with experimental results (see also Computational Details section in the main text).

[b] $A_\parallel$ is defined as the component of the effective HFC components with the largest difference to $A_{iso} = (A_x + A_y + A_z)/3$ (Table 2), and $A_\perp$ the average of those components with the smallest difference to A_{iso}.

[c] The on-site hyperfine values –a_x, a_y, a_z– of Mn$_i$ are derived from: $a_x = A_{BS,x} \cdot \frac{\langle S_z \rangle_{BS}}{S_i} \cdot 1.78$, and $a_{iso} = (a_x + a_y + a_z)/3$.

[b] a_{aniso} is defined as $a_{aniso} = a_\perp - a_\parallel$, where $a_\parallel$ the on-site HFC component with the largest difference to a_{iso} and $a_\perp$ the average of those components with the smallest difference to a_{iso}.

Table S5. Calculated effective/projected ^{14}N hyperfine and NQI tensors (in MHz) for the electron–nuclear couplings of the bound NH_x-N and of the His332 imino-N, and ^{17}O hyperfine tensors (in MHz) of the terminal W1 and W2 ligands and the bridging O5 ligand for the S_2-NH_3 and the S_2-state models in the low-spin ($S = 1/2$) configuration.

		^{14}N		^{17}O		
		NH_3	His332	O5	W2	W1
**A4**	$A_{BS,x}$ [a]	3.64	-10.76		15.04	2.18
	$A_{BS,y}$	8.12	-13.25		-15.40	-3.04
	$A_{BS,z}$	9.96	-14.26		-62.54	-15.96
	$\|A_{iso}\|$ [b]	3.06	3.42	-	16.66	3.80
	A_{dip} [c]	0.76	0.27	-	-8.48	-2.20
	A_{η} [d]	0.51	0.51	-	0.01	0.10
	$\|e^2Qq/h\|$	1.44	1.96			
	η	0.81	0.85			
**A5**	$A_{BS,x}$	6.64	-2.38		0.97	-0.18
	$A_{BS,y}$	14.86	-6.17		-1.25	-1.76
	$A_{BS,z}$	-16.96	-6.89		-4.23	-6.31
	$\|A_{iso}\|$	6.43	2.49	-	0.56	0.72
	A_{dip}	1.55	0.67	-	-0.27	-0.47
	A_{η}	0.34	0.26	-	0.13	0.45
	$\|e^2Qq/h\|$	0.8	2.23			
	η	0.88	0.60			
**A8**	$A_{BS,x}$	5.66	-8.10		1.09	-1.93
	$A_{BS,y}$	-9.59	-11.39		-1.22	-4.41
	$A_{BS,z}$	25.74	-12.65		-39.93	-6.27
	$\|A_{iso}\|$	3.61	5.18	-	3.87	1.16
	A_{dip}	-1.60	0.63	-	-3.55	0.31
	A_{η}	0.33	0.48	-	0.01	0.81
	$\|e^2Qq/h\|$	0.87	2.23			
	η	0.97	0.50			
**A9**	$A_{BS,x}$	-12.35	-8.82		-0.13	-1.71
	$A_{BS,y}$	12.91	-11.89		1.81	-3.81
	$A_{BS,z}$	23.75	-13.04		-3.97	-6.42
	$\|A_{iso}\|$	3.59	5.55	-	0.44	0.89
	A_{dip}	-0.81	0.60	-	-0.22	-0.27
	A_{η}	0.08	0.47	-	0.84	0.86
	$\|e^2Qq/h\|$	0.62	2.16			
	η	0.96	0.56			

**A10**	$A_{BS,x}$	1.53	-11.81		0.45	-2.50
	$A_{BS,y}$	6.51	-14.00		1.05	-30.93
	$A_{BS,z}$	9.12	-15.04		-1.49	-30.93
	$\|A_{iso}\|$	2.35	3.45	-	0.54	8.18
	A_{dip}	0.86	0.23	-	0.15	-4.33
	A_{η}	0.62	0.57	-	0.82	0.57
	$\|e^2Qq/h\|$	1.33	1.86			
	η	0.80	0.82			
**B1**	$A_{BS,x}$	-7.73	-9.65	6.62	10.71	
	$A_{BS,y}$	-7.90	-12.54	28.15	-11.25	
	$A_{BS,z}$	-10.23	-13.69	-40.78	-52.28	
	$\|A_{iso}\|$	3.03	5.44	8.14	4.66	-
	A_{dip}	-0.28	0.52	3.00	-2.47	-
	A_{η}	0.10	0.50	0.68	0.24	-
	$\|e^2Qq/h\|$	1.27	2.09			
	η	0.77	0.71			
**B3**	$A_{BS,x}$	-7.54	-10.34	-10.28	1.41	
	$A_{BS,y}$	-7.57	-12.97	28.88	2.46	
	$A_{BS,z}$	-9.87	-14.04	-47.07	-6.01	
	$\|A_{iso}\|$	3.53	5.21	10.79	1.40	-
	A_{dip}	-0.33	0.44	3.46	-0.58	-
	A_{η}	0.02	0.51	0.99	0.39	-
	$\|e^2Qq/h\|$	1.29	2.00			
	η	0.91	0.79			
**C1**	$A_{BS,x}$	-5.88	-9.46	20.12	17.00	
	$A_{BS,y}$	-6.10	-12.43	26.68	-22.23	
	$A_{BS,z}$	-6.21	-13.54	-40.98	-91.64	
	$\|A_{iso}\|$	1.23	5.87	11.87	-	8.82
	A_{dip}	0.02	0.58	-2.38	-	-4.85
	A_{η}	0.61	0.47	0.56	-	0.11
	$\|e^2Qq/h\|$	1.84	2.12			
	η	0.30	0.70			
**C2**	$A_{BS,x}$	-7.78	-10.31	-17.19	0.98	
	$A_{BS,y}$	-7.97	-12.96	32.91	-4.03	
	$A_{BS,z}$	-9.03	-14.01	-43.88	-17.53	
	$\|A_{iso}\|$	3.71	4.93	12.23	-	3.38
	A_{dip}	-0.17	0.42	2.76	-	-2.25

	A_{η}	0.25	0.50	0.78	-	0.30
	$\|e^2Qq/h\|$	1.68	2.00			
	η	0.34	0.79			
**D4**	$A_{BS,x}$	-8.17	-0.24	4.85	10.47	-41.35
	$A_{BS,y}$	-8.41	-4.11	-26.54	-11.25	-44.09
	$A_{BS,z}$	-11.09	-4.73	27.70	-51.66	-76.52
	$\|A_{iso}\|$	4.64	0.95	8.22	12.29	16.84
	A_{dip}	-0.47	0.43	3.10	-6.84	-3.51
	A_{η}	0.12	0.22	0.08	0.03	0.12
	$\|e^2Qq/h\|$	1.23	2.23			
	η	0.90	0.59			
**D5**	$A_{BS,x}$	5.21	-7.28	-50.40	1.48	2.27
	$A_{BS,y}$	5.54	-10.76	-94.61	-1.95	-10.72
	$A_{BS,z}$	8.33	-11.44	-115.06	-5.70	21.23
	$\|A_{iso}\|$	1.99	3.60	33.89	0.95	3.56
	A_{dip}	-0.31	0.47	7.09	-0.41	-1.54
	A_{η}	0.17	0.27	0.56	0.18	0.86
	$\|e^2Qq/h\|$	0.98	2.10			
	η	0.73	0.71			
**E1**	$A_{BS,x}$	23.73	2.06	27.36	10.69	17.70
	$A_{BS,y}$	23.82	-2.25	-30.07	-25.77	-20.79
	$A_{BS,z}$	35.03	-2.85	36.09	-67.39	-67.36
	$\|A_{iso}\|$	13.68	1.18	4.77	5.35	5.46
	A_{dip}	-1.86	-0.11	-0.38	-2.53	-2.48
	A_{η}	0.01	0.40	0.55	0.46	0.10
	$\|e^2Qq/h\|$	2.89	2.48			
	η	0.31	0.47			
**E2**	$A_{BS,x}$	-5.21	25.31	0.57	10.66	3.46
	$A_{BS,y}$	-7.26	25.82	-26.76	-10.80	-5.85
	$A_{BS,z}$	-8.22	32.01	38.26	-51.07	6.26
	$\|A_{iso}\|$	3.15	10.21	8.60	11.06	2.37
	A_{dip}	0.39	-0.79	4.19	-5.51	0.47
	A_{η}	0.57	0.12	0.54	0.01	0.20
	$\|e^2Qq/h\|$	1.73	3.37			
	η	0.72	0.18			
**E3**	$A_{BS,x}$	23.85	0.30	-14.51	-1.35	-1.55
	$A_{BS,y}$	23.94	-3.69	-36.67	-2.54	2.29

	$A_{\mathrm{BS},z}$	34.10	-4.24	39.40	-7.89	-13.57
	$\lvert A_{\mathrm{iso}}\rvert$	10.56	1.06	11.54	1.73	2.55
	A_{dip}	-1.32	0.47	3.00	-0.87	-1.70
	A_{η}	0.01	0.22	0.17	0.30	0.10
	$\lvert e^2Qq/h\rvert$	2.94	2.27			
	η	0.19	0.61			
**S_2**	$A_{\mathrm{BS},x}$		-9.80	2.36	9.93	1.37
	$A_{\mathrm{BS},y}$		-12.66	36.65	-10.16	3.71
	$A_{\mathrm{BS},z}$		-13.81	-37.39	-56.84	-5.09
	$\lvert A_{\mathrm{iso}}\rvert$		5.38	8.53	9.45	1.25
	A_{dip}		0.51	3.87	-5.75	0.37
	A_{η}		0.50	0.03	0.01	0.68
	$\lvert e^2Qq/h\rvert$		2.08			
	η		0.72			
**S_2^{H}**	$A_{\mathrm{BS},x}$		-10.48	-14.56	-0.17	1.34
	$A_{\mathrm{BS},y}$		-13.09	35.52	0.75	-2.75
	$A_{\mathrm{BS},z}$		-14.15	-44.25	-7.63	-13.26
	$\lvert A_{\mathrm{iso}}\rvert$		5.14	12.02	1.24	2.52
	A_{dip}		0.43	3.23	-1.04	-2.27
	A_{η}		0.51	0.52	0.12	0.14
	$\lvert e^2Qq/h\rvert$		1.99			
	η		0.80			

[a] $A_{\mathrm{BS},x}$, $A_{\mathrm{BS},y}$, $A_{\mathrm{BS},z}$ are the components of the hyperfine coupling tensor computed from the BS calculation and they follow the convention $|A_{\mathrm{BS},z}| > |A_{\mathrm{BS},y}| > |A_{\mathrm{BS},x}|$. The effective (observable) HFC components –A_x, A_y, A_z– of ^{14}N or ^{17}O ligand to Mn$_i$ are derived from: $A_x = A_{\mathrm{BS},x} \cdot \frac{\langle S_z \rangle_{\mathrm{BS}}}{S_i} \cdot \rho_i$, where S_i is the site spin of Mn$_i$ [2 for Mn(III) and 3/2 for Mn(IV)], $\langle S_z \rangle_{\mathrm{BS}}$ the total M_S of the BS wavefunction (1/2 in all models), and ρ_i the spin projection coefficient of Mn$_i$. For a bridging ligand coordinated to Mn$_j$ and Mn$_k$, such as NH$_x$ in models A and O5, the effective HFC components are derived from $A_x = \frac{A_{\mathrm{BS},x}}{2} \cdot (\frac{\langle S_z \rangle_{\mathrm{BS}}}{S_j} \cdot |\rho_j| + \frac{\langle S_z \rangle_{\mathrm{BS}}}{S_k} \cdot |\rho_k|)$, where S_j and ρ_j are the site spin and the spin projection coefficient, respectively, of Mn$_j$.[1-3]

[b] $|A_{\mathrm{iso}}|$ is defined as the average of the principal components of the effective hyperfine tensor: $|A_{\mathrm{iso}}| = (|A_x| + |A_y| + |A_z|)\,/3$.

[c] A_{dip} is defined as $A_{\mathrm{dip}} = (T_1 + T_2)/2 = -T_3/2$, where T_1, T_2, and T_3 represent the three principal components of the hyperfine tensors minus A_{iso}, i.e. $|A_x| - |A_{\mathrm{iso}}|$, $|A_y| - |A_{\mathrm{iso}}|$, and $|A_z| - |A_{\mathrm{iso}}|$, respectively, and are labeled such that $|T_1| \le |T_2| \le |T_3|$. [d] The rhombicity is defined as $A_\eta = (T_1 - T_2)/T_3$, respectively.

Table S6. Electron affinity (EA) differences between S_2-NH_3 and the S_2-state models, i.e. ΔE = EA(S_2) – EA(S_2-NH_3). Comparisons are made between structures having the same charge, thus models **A5**, **B1**, **C1**, **D4**, and **E2** are compared with $\mathbf{S_2}$ and models **A4**, **A9**, **B3**, **C2**, **D5**, and **E3** are compared with $\mathbf{S_2^H}$.

		ΔE (kcal mol^{-1})
S_2	**A5**	8.73
	**B1**	2.19
	**C1**	4.43
	**D4**	2.39
	**E2**	0.68
S_2^H	**A4**	0.34
	**A9**	9.87
	**B3**	1.57
	**C2**	1.97
	**D5**	3.33
	**E3**	2.90

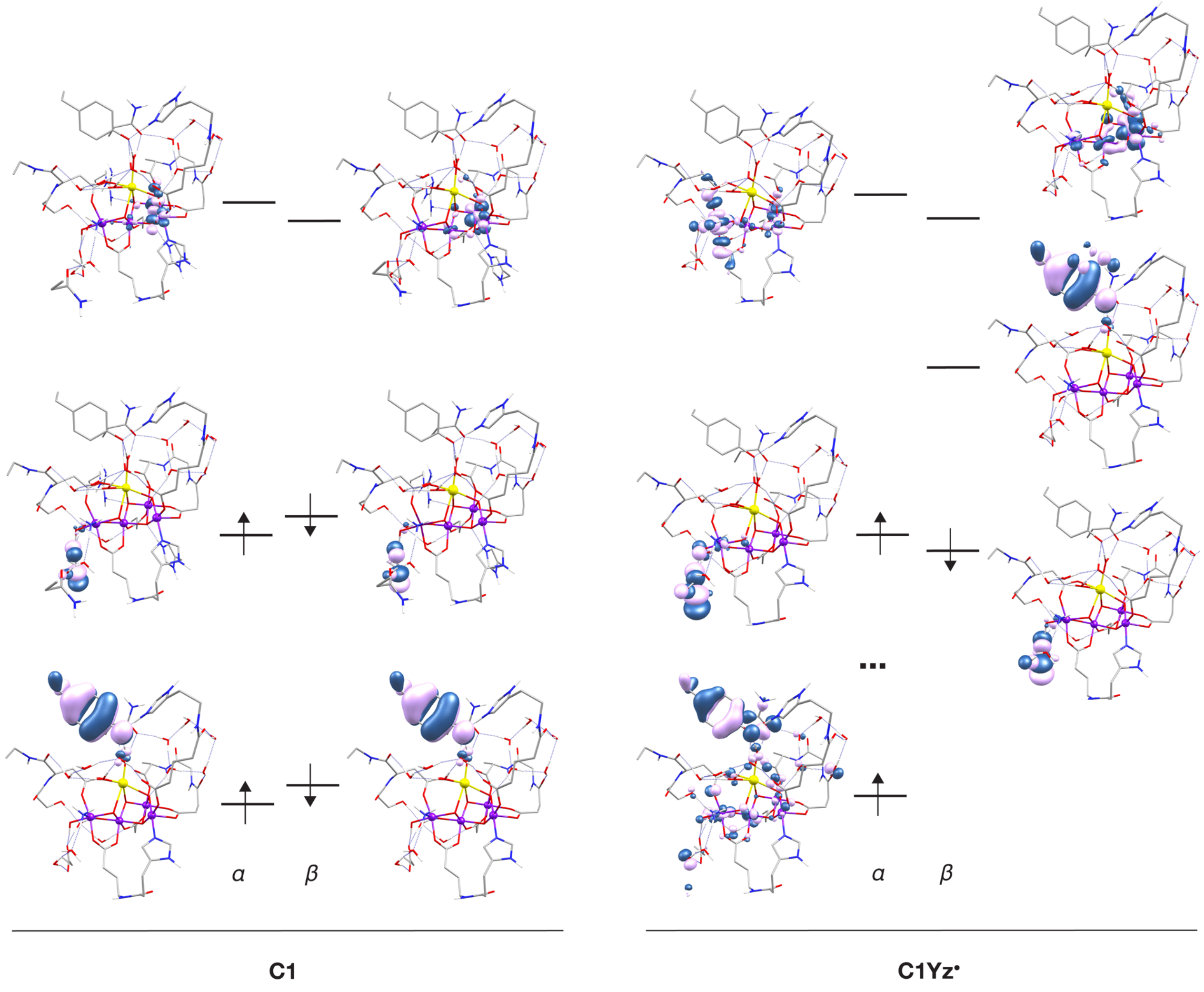

Figure S2. Frontier orbitals of model **C1** of the ammonia-bound S_2 state of the OEC (left) and of the oxidized **C1Y$_z^•$** (right); hydrogen atoms are omitted for clarity.

Table S7. Comparison of calculated ^{55}Mn HFCs (MHz) for the model in which ammonia binds on the W1 position (**B1**) between this work, Schraut and Kaupp,[4] and Lohmiller et al.[5]

	A_1	A_2	A_3	A_4	A_1/A_2	A_2/A_3	A_3/A_4
**B1**	311 [1]	291 [4]	248 [2]	206 [3]	1.07	1.17	1.21
**Ref 4**	295 [1]	192 [2]	160 [4]	120 [3]	1.54	1.20	1.33
**Ref 5**	244 [1]	227 [4]	215 [2]	166 [3]	1.07	1.06	1.30

Table S8. Comparison of calculated ^{14}N HFCs and NQI asymmetry (η) for **B1** between this work, Schraut and Kaupp,[4] and Lohmiller et al.[5]

	NH_3		His332	
	$\|A_{iso}\|$	η	$\|A_{iso}\|$	η
**B1**	3.03	0.77	5.44	0.71
**Ref 4**	3.39	0.35	5.67	0.94
**Ref 5**	2.68	0.87	6.10	0.88

Orca Input files

Broken-Symmetry:

```
! UKS TPSSh RIJCOSX ZORA ZORA-TZVP SARC/J
! Grid5 GridX7 NoFinalGrid TightSCF moread

%pal nprocs 10 end
%maxcore 3000

%moinp "filename.gbw"

%basis newgto C "ZORA-SVP" end
       newgto H "ZORA-SVP" end
end

%method intacc 6.0 end

%scf maxiter 200
     shift shift 0.10 erroff 0.1 end
     FlipSpin 1,2 FinalMs 0.5
end


*xyzfile 1 14 filename.xyz
```

Mn hyperfine coupling tensors:

```
! UKS TPSSh RIJCOSX ZORA ZORA-def2-TZVP(-f) SARC/J
! Grid5 GridX7 NoFinalGrid Slowconv TightSCF moread

%pal nprocs 10 end
%maxcore 15000

%moinp "filename.gbw"

%basis newgto C "ZORA-def2-SVP" end
       newgto H "ZORA-def2-SVP" end
       newgto Mn
 S 1
   1 4331015.6489100000      1.0000000000
 S 1
   1 1732406.2595600000      1.0000000000
 S 1
   1  692962.5038250000      1.0000000000
 S 1
   1  277185.0015300000      1.0000000000
 S 1
   1   41550.7698900000      1.0000000000
 S 1
   1    9455.9700152000      1.0000000000
 S 1
   1    2676.5206482000      1.0000000000
 S 1
   1     871.4668753000      1.0000000000
 S 1
   1     312.9830642000      1.0000000000
 S 1
   1     121.4445405100      1.0000000000
 S 1
   1      47.9225988300      1.0000000000
 S 1
   1     303.6672316300      1.0000000000
```

```
 S 1
   1      93.8814031900      1.0000000000
 S 1
   1      14.8794212100      1.0000000000
 S 1
   1       6.2865200700     -1.0000000000
 S 1
   1       9.4858591300      1.0000000000
 S 1
   1       1.5698706200      1.0000000000
 S 1
   1       0.6590321400     -1.0000000000
 S 1
   1       0.1068629200     -1.0000000000
 S 1
   1       0.0392674400     -1.0000000000
 P 6
   1    1444.7978182000      0.0032493630
   2     342.0655119700      0.0237346473
   3     109.5840089100      0.1067232634
   4      40.7479881700      0.3007617717
   5      16.1886265700      0.4790147038
   6       6.5484506000      0.2708283465
 P 1
   1      25.3570864400     -1.0000000000
 P 1
   1       3.4830168800      1.0000000000
 P 1
   1       1.3858800900      1.0000000000
 P 1
   1       0.5255509500     -1.0000000000
 P 1
   1       0.1276500000      1.0000000000
 D 4
   1      56.5631891200      0.0192444440
   2      16.2787347100      0.1161060412
   3       5.6964273900      0.3706567953
   4       2.1411147900      0.6570040408
 D 1
   1       0.7829180200      1.0000000000
 D 1
   1       0.2595231100      1.0000000000
 D 1
   1       0.0860000000      1.0000000000
 F 1
   1       1.3260000000      1.0000000000
 end
end

%method intacc 6.0
        SpecialGridAtoms 25
        SpecialGridIntAcc 11
end

%rel picturechange true end

%scf maxiter 200
     shift shift 0.10 erroff 0.1 end
     CNVSOSCF true SOSCFstart 0.001
end

*xyzfile 1 2 filename.xyz

%eprnmr   nuclei = all Mn { aiso, adip, aorb }
end
```

N and O hyperfine coupling tensors:

```
! UKS TPSSh RIJCOSX ZORA ZORA-def2-TZVP(-f) SARC/J
! Grid5 GridX7 NoFinalGrid Slowconv TightSCF moread

%pal nprocs 10 end
%maxcore 15000

%moinp "filename.gbw"

%basis
 NewGTO N
 S 1
   1   308293.7601093750      1.0000000000
 S 1
   1   123317.5040437500      1.0000000000
 S 1
   1    49327.0016175000      1.0000000000
 S 1
   1    19730.8006470000      1.0000000000
 S 1
   1     2957.8958745000      1.0000000000
 S 1
   1      673.2213359500      1.0000000000
 S 1
   1      190.6824949400      1.0000000000
 S 1
   1       62.2954419000      1.0000000000
 S 1
   1       22.6541611800      1.0000000000
 S 1
   1        8.9791477400      1.0000000000
 S 1
   1        3.6863002400      1.0000000000
 S 1
   1        0.8466007700      1.0000000000
 S 1
   1        0.3364713400      1.0000000000
 S 1
   1        0.1364765400      1.0000000000
 P 4
   1       49.2003805100      0.0115131336
   2       11.3467905400      0.0785386070
   3        3.4273972400      0.3081978103
   4        1.1785525100      0.7201896814
 P 1
   1        0.4164220500      1.0000000000
 P 1
   1        0.1426082600      1.0000000000
 D 1
   1        1.6540000000      1.0000000000
 D 1
   1        0.4690000000      1.0000000000
 end
NewGTO O
 S 1
   1   422380.9786093750      1.0000000000
 S 1
   1   168952.3914437500      1.0000000000
 S 1
   1    67580.9565775000      1.0000000000
 S 1
   1    27032.3826310000      1.0000000000
 S 1
```

```
   1     4052.3871392000      1.0000000000
 S 1
   1      922.3272271000      1.0000000000
 S 1
   1      261.2407098900      1.0000000000
 S 1
   1       85.3546413500      1.0000000000
 S 1
   1       31.0350352400      1.0000000000
 S 1
   1       12.2608607300      1.0000000000
 S 1
   1        4.9987076000      1.0000000000
 S 1
   1        1.1703108200      1.0000000000
 S 1
   1        0.4647474100      1.0000000000
 S 1
   1        0.1850453600      1.0000000000
 P 4
   1       63.2749548000      0.0121096626
   2       14.6270493800      0.0831734399
   3        4.4501223500      0.3200012219
   4        1.5275799600      0.7069790865
 P 1
   1        0.5293511800      1.0000000000
 P 1
   1        0.1747842100      1.0000000000
 D 1
   1        2.3140000000      1.0000000000
 D 1
   1        0.6450000000      1.0000000000
 end
end

%method intacc 6.0
        SpecialGridAtoms 8,7
        SpecialGridIntAcc 9,9
end

%rel
     SOCFlags 1,3,3,1
     picturechange true
end

%scf maxiter 200
     shift shift 0.10 erroff 0.1 end
     CNVSOSCF true SOSCFstart 0.001
end

*xyzfile 1 2 filename.xyz

%eprnmr
   nuclei = 10 { aiso, adip, aorb, fgrad }
   nuclei = 11 { aiso, adip, aorb, fgrad }
   nuclei = 12 { aiso, adip, aorb, fgrad }
   nuclei = 83 { aiso, adip, aorb, fgrad }
end
```

References

(1) Pantazis, D. A.; Orio, M.; Petrenko, T.; Zein, S.; Bill, E.; Lubitz, W.; Messinger, J.; Neese, F. A New Quantum Chemical Approach to the Magnetic Properties of Oligonuclear Transition-Metal Complexes: Application to a Model for the Tetranuclear Manganese Cluster of Photosystem II. *Chem. Eur. J.* **2009**, *15*, 5108-5123.

(2) Cox, N.; Ames, W.; Epel, B.; Kulik, L. V.; Rapatskiy, L.; Neese, F.; Messinger, J.; Wieghardt, K.; Lubitz, W. Electronic Structure of a Weakly Antiferromagnetically Coupled $Mn^{II}Mn^{III}$ Model Relevant to Manganese Proteins: A Combined EPR, ^{55}Mn-ENDOR, and DFT Study. *Inorg. Chem.* **2011**, *50*, 8238-8251.

(3) Rapatskiy, L.; Ames, W. M.; Pérez-Navarro, M.; Savitsky, A.; Griese, J. J.; Weyhermüller, T.; Shafaat, H. S.; Högbom, M.; Neese, F.; Pantazis, D. A.; Cox, N. Characterization of Oxygen Bridged Manganese Model Complexes Using Multifrequency ^{17}O-Hyperfine EPR Spectroscopies and Density Functional Theory. *J. Phys. Chem. B* **2015**, *119*, 13904-13921.

(4) Schraut, J.; Kaupp, M. On Ammonia Binding to the Oxygen-Evolving Complex of Photosystem II: A Quantum Chemical Study. *Chem. Eur. J.* **2014**, *20*, 7300-7308.

(5) Lohmiller, T.; Krewald, V.; Navarro, M. P.; Retegan, M.; Rapatskiy, L.; Nowaczyk, M. M.; Boussac, A.; Neese, F.; Lubitz, W.; Pantazis, D. A.; Cox, N. Structure, ligands and substrate coordination of the oxygen-evolving complex of photosystem II in the S_2 state: a combined EPR and DFT study. *Phys. Chem. Chem. Phys.* **2014**, *16*, 11877-11892.
